# A bioprocess engineering approach for the production of hydrocarbons and fatty acids from green microalga under high cobalt concentration as the feedstock of high-grade biofuels

**DOI:** 10.1186/s13068-024-02512-6

**Published:** 2024-05-10

**Authors:** Alok Patel, Chloe Rantzos, Eleni Krikigianni, Ulrika Rova, Paul Christakopoulos, Leonidas Matsakas

**Affiliations:** Biochemical Process Engineering, Division of Chemical Engineering, Department of Civil, Environmental and Natural Resources Engineering, Luleå University of Technology, 971 87 Luleå, Sweden

**Keywords:** Biofuels, Hydrocarbons, Fatty acids, Pyrolysis, Open raceway pond, *Botryococcus braunii*

## Abstract

*Botryococcus braunii*, a colonial green microalga which is well-known for its capacity to synthesize hydrocarbons, has significant promise as a long-term source of feedstock for the generation of biofuels. However, cultivating and scaling up *B. braunii* using conventional aqua-suspended cultivation systems remains a challenge. In this study, we optimized medium components and light intensity to enhance lipid and hydrocarbon production in a multi-cultivator airlift photobioreactor. BBM 3N medium with 200 μmol/m^2^/s light intensity and a 16 h light–8 h dark regimen yielded the highest biomass productivity (110.00 ± 2.88 mg/L/day), as well as the highest lipid and hydrocarbon content. Cultivation in a flat-panel bioreactor resulted in significantly higher biomass productivity (129.11 ± 2.74 mg/L/day), lipid productivity (32.21 ± 1.31 mg/L/day), and hydrocarbon productivity (28.98 ± 2.08 mg/L/day) compared to cultivation in Erlenmeyer flasks and open 20-L raceway pond. It also exhibited 20.15 ± 1.03% of protein content including elevated levels of chlorophyll *a*, chlorophyll *b*, and carotenoids. This work is noteworthy since it is the first to describe fatty acid and hydrocarbon profiles of *B. braunii* during cobalt treatment. The study demonstrated that high cobalt concentrations (up to 5 mg/L of cobalt nitrate) during *Botryococcus* culture affected hydrocarbon synthesis, resulting in high amounts of *n*-alkadienes and trienes as well as lipids with elevated monounsaturated fatty acids concentration. Furthermore, pyrolysis experiments on microalgal green biomass and de-oiled biomass revealed the lipid and hydrocarbon compounds generated by the thermal degradation of *B. braunii* that facilitate extra economical value to this system.

**Supplementary Information:**

The online version contains supplementary material available at 10.1186/s13068-024-02512-6.

## Introduction

Population growth and the consequent demand for resources render the need for innovative and sustainable solutions. A critical area is the sustainable production of food, fuel, and other consumables [1]. Microalgae are found in almost every aquatic environment. They include eukaryotic microorganisms and prokaryotic cyanobacteria (blue-green algae), encompassing more than 25,000 species [2]. Since ancient civilizations, microalgae have been used as a source of nutrition for humans but became the subject of scientific studies at the end of the nineteenth century [3]. Lately, they have gained increasing interest owing to their potential for mitigating CO_2_ emissions, as well as biofuel production and value-added products [4]. Algae can rapidly synthesize substantial quantities of lipids, proteins, and carbohydrates [5]. Particularly, certain microalgal species can accumulate lipids comprising up to 90% of their dry biomass. The lipid composition of microalgae, however, fluctuates based on factors like species, cultivation conditions, and growth stage [6]. Microalgal lipids possess an appealing quality as a biofuel feedstock, particularly for biodiesel, due to their energy-dense C–C and C–H bonds. Algal oil is converted to its corresponding fatty ester through transesterification reaction. Microalgae-derived hydrocarbons are another desirable feedstock for biomass conversion into liquid biofuel, which can then undergo hydrocracking to produce gasoline or be mixed directly with aviation fuel [7]. Due to the significant energy requirements associated with the transesterification process, which can contribute to as much as 70% to 80% of the inclusive cost of biodiesel production, microalgal hydrocarbons are particularly attractive as they avoid the need for such reaction. The extraction of the final transesterification product, besides being expensive, it often requires the use of harmful catalysts, and has an outsized impact on the economic viability of biofuel production [8].

The single-celled photosynthetic microalga belonging to the Chlorophyceae subgroup within the Chlorophyta phylum *Botryococcus braunii* has great potential as a source of sustainable biomass, a liquid hydrocarbons reservoir, while gathering extended-chain hydrocarbons (comprising up to 75% of its dry biomass) as well as ether lipids [9].

The three known races of *B. braunii* differ on the basis of the prevalent hydrocarbon they produce [10]. Race A usually produced C25–C31, *n*-alkadienes and alkatrienes, mainly odd numbered, that can be up to 61% based on dry weight in active colonies. On the other hand, Race B is known as a source of botryococcenes, which are polymethylated unsaturated triterpenes (C_n_H_2n–10_, with n ranging from 30 to 37), and can reach amounts between 27 to 86% on the cell dry weight basis [11]. Finally, Race L synthesizes lycopadiene (C_40_H_78_), a tetraterpene accounting from 2 to 8% of dry biomass. During active growth, all three strains achieve a green coloration, while in the stationary phase the color of the colonies varies from red–orange (race B) to orange-brownish (race L) and pale yellow (race A) [11].

To fully harness *B. braunii’*s competence for sustainable hydrocarbon synthesis, it is crucial to focus on increasing biomass yields and improving hydrocarbon productivity. While nutritional requirements and culture conditions have been optimized for hydrocarbon production, the effect of these factors on the lipids profile of this microorganism remains poorly understood. Cobalt, a vital transition metal widely utilized in the aerospace industry, electronics industries, cemented carbide, insulators, catalysts at industrial scale, and ceramics poses a significant concern to the environment. Improperly treated and discharged cobalt-rich streams can result in severe harm to animals and humans [12]. Conventional approaches to mitigate heavy metal toxicity have proven ineffective or prohibitively expensive when dealing with cobalt concentrations below a certain threshold (< 10 mg/L). Interestingly, certain species of *Botryococcus* not only exhibit high tolerance to metallic cobalt concentrations, but this triggers also a 20% increase in extracellular hydrocarbon content [13]. The current study aimed to investigate the growth of *B. braunii* SAG 807–1 (Race A) under elevated cobalt concentrations using three distinct cultivation strategies, namely Erlenmeyer flasks, flat-panel photobioreactors, and open raceway pond. The objective was to assess the biomass yield, as well as the quantity and composition of lipids and hydrocarbons produced. Flasks are typically used for laboratory-scale cultivation of microalgae for screening purposes or small-scale experiments, being inexpensive and easy to use, but their small size limits the amount of biomass that can be produced. Photobioreactors are closed systems that allow for precise control of temperature, light intensity, and CO_2_ concentration. They can be designed to optimize microalgal growth and productivity and are used for large-scale production. Although photobioreactors are more expensive to set up and used for cultivation purposes than open ponds, they offer greater control over environmental conditions and can achieve higher yields of algal biomass [14]. Open ponds are more straightforward systems and used for large-scale microalgae cultivation. These ponds are configured in a raceway, where a paddlewheel facilitates the circulation and mixing of cultures and nutrients. They are more cost-effective and easier to operate than photobioreactors and can generate high biomass yields. However, their productivity and efficiency can be affected by weather conditions, while contamination by other microorganisms is another potential drawback [15].

After processing the microalgal biomass for the removal of lipids and other extractables, around half of the initial biomass remains as a residue, as lipids take up to 55% of total cell dry weight. This residual biomass can be used for biogas production through anaerobic digestion and for soil amelioration in agriculture. However, even the lipid-free biomass consisting mainly of protein and carbohydrates, provides a valuable source of biofuel. Pyrolysis is a way to convert this residue into bio-oil after the extraction of lipids. Three major streams are generated after pyrolysis of microalgal biomass: a condensed liquid known as bio-oil, gaseous products, and biochar. Based on microalgal species, growth and reaction conditions, the final product varies in terms of composition, containing bio-oil (18% to 58 wt%), water-soluble compounds (15–30 wt%), gases (10–60 wt%), and biochar (15–43 wt%), respectively [16]. In theory, owing to its simpler composition, the lipid-free microalgal residue would require a lower energy input during pyrolysis than raw biomass [17]. According to Vardon et al., the bio-oil yield was only 7% lower when using lipid-free *Scenedesmus* residues compared to raw algal biomass [18]. Integrating the pyrolysis of de-oiled microalgal biomass with fatty acids removal could result in a 43% increase in the overall oil yield, as compared to the direct pyrolysis of microalgae alone [17]. In the current study, we attempted the pyrolysis of freeze-dried biomass (green material), the hydrocarbon-rich white portion of biomass and de-oiled biomass, to assess their suitability for bio-oil production.

Along with their ability to accumulate lipids and hydrocarbons, under stress-inducing conditions, certain microalgal species synthesize distinct secondary metabolites, including pigments and vitamins, which result in added-value products with industrial applications, such as cosmetics, food, and pharmaceuticals [19]. Several microalgal strains are promising sources of protein, with some strains having a comparable content to that of meat, eggs, soybean, and milk [20]. Their use as an alternative feedstock for biofuels can help decrease the dependence on fossil fuels, while also reducing greenhouse gas emissions and mitigating the effects of climate change. Moreover, microalgae are advantageous over traditional biofuel feedstocks in terms of cultivation resources as they promote the sustainable use of land, while tackling wastewater treatment problems and pollution of water bodies. Evidently, microalgae hold a crucial role in the efforts towards a greener manufacturing of bioproducts and biofuels.

In this study, we have established novel ground in integrating the development of this strain under a variety of culture techniques, such as shake flasks, a photobioreactor, and an indoor open race pond. This all-encompassing method enables us to examine its biochemical properties, which include proteins, hydrocarbons, lipids, pigments, and more. Additionally, we investigated the possible uses of de-oiled and dehydrated biomass and considered the viability of pyrolyzing it to produce bio-oil. This holistic approach advances our knowledge of *Botryococcus* significantly and provides chances to explore novel approaches for the production of biofuel.

## Material and methods

### Microalgal cultivation

*Botryococcus braunii* SAG 807-1 was sourced from the Sammlung von Algenkulturen (SAG) culture collection of Algae at Göttingen University, Germany. The culture of *B. braunii* was then transferred to flasks with modified Bold's basal medium (BBM; B5282, Sigma-Aldrich) grown under photoautotrophic conditions at 25 ℃*.* Prior to inoculation, all cultivation flasks/tubes underwent autoclaving using the Tuttnauer 5075 ELVC-G-D to ensure sterility. The inoculation process was conducted in a laminar airflow environment (Telstar BiOptima 3) to maintain sterile conditions. Glycerol stocks prepared in a similar medium were used as inocula for subsequent test cultivation.

### Batch cultivation of *Botryococcus braunii* photoautotrophically

Cultivations were performed initially in a multi-cultivator photobioreactor (MC 1000-OD; PSI, Czech Republic) equipped with eight cylindrical tubes. Each tube was connected with an aeration tube to supply air at normal pressure and the light intensity was adjusted at 100 μmol/m^2^/s with a 16-h light and 8-h dark régime. The temperature was adjusted with an external chiller at 25 ℃. Three different media compositions, BBM, BG11 and Chu modified, were used for biomass optimization. BBM and BG11 were purchased as a concentrated solution of 50 × and 100 × from commercial sources (Sigma Aldrich, USA) while Chu modified medium was prepared by mixing individual ingredients according to Furuhashi et al. [21]. To assess the impact of nitrogen concentration on growth and lipid production, *B. braunii* was cultivated in BBN 2N or BBN 3N, which contained 2 × and 3 × the amount of sodium nitrate in BBM. After determining the optimal medium composition, the light intensity was altered from 50 μmol/m^2^/s to 600 μmol/m^2^/s in both continuous mode of light and a 16/8-h light and dark photoperiod to optimize biomass, lipid, and hydrocarbon production.

### Cultivation in flasks and a flat-panel photobioreactor

Besides mini-cultivators, the microalga was also cultivated in 1-L Erlenmeyer flasks placed in an incubator shaker and a 1.9-L flat-panel airlift-photobioreactor (Labfors 5; Infors AG, Basel, Switzerland). In the former, the light intensity was maintained at around 180–200 µmol/m^2^/s with external warm light LED strips; whereas in the latter, it varied to 200 µmol/m^2^/s by an internal LED lights under a 16/8-h light/dark photoperiod.

### Scale-up of cultivation to a 20-L open raceway pond

**Fig. 1:**
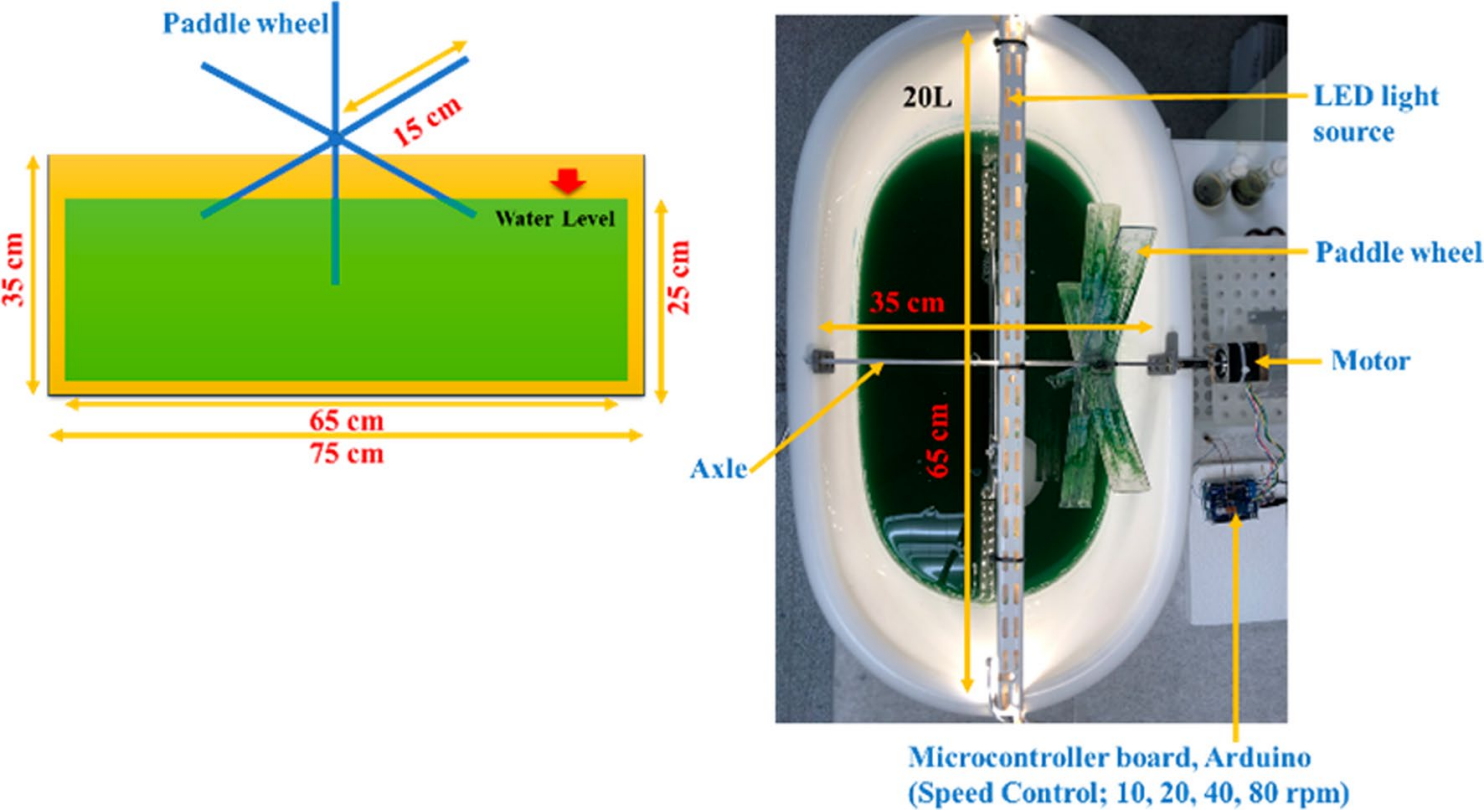
Side and top view of the open raceway pond, with details of the paddle wheel driven by a speed motor controlled by Arduino software

The primary objective of the open raceway pond design was to maximize the exposure of microalgae to illumination. To prevent shadowing, which hinders the access of light into the growth medium and reduces illumination of cells in the lower water column, a depth of 0.3 m was chosen [22]. Circulation of the microalgae culture in the 25-L open raceway pond was facilitated by a paddle wheel driven by a speed motor (RS PRO Hybrid Stepper Motor, 12 V, 1.8°, 42.3 × 42.3 mm frame, 5 mm shaft; RS, Fort Worth, TX, USA) controlled by Arduino software (Uno Rev 3, 715–4081; RS) (Fig. 1). The speed of rotation of the plastic paddle wheel was regulated to 10–80 rpm to generate fluid movement. The paddle wheel was made of six identical blades, each having dimensions of 150 × 50 × 3.5 mm. The rotation of the paddle wheel ensured adequate nutrient and light distribution throughout the culture, providing optimal growth and productivity of the microalgae. The lighting system in the setup was specifically designed to maintain a light intensity of 200 ± 20 μmol/m^2^/s, which supported microalgal photosynthetic activity and growth. The photoperiod followed a 16 h light–8 h dark cycle.

### Effect of cobalt stress on the synthesis of hydrocarbons and lipids by *B. braunii* cultivated under photoautotrophic conditions

In the first stage of the experiment, the microalga was grown on BBM in mini-photobioreactors to investigate the impact of different concentrations (0.49–50 mg/L) of cobalt nitrate on the generation of biomass, lipids, and hydrocarbons. Based on the results obtained from the mini-photobioreactor experiments, the optimal concentration of cobalt nitrate was identified and used for further experiments in other vessels. To enhance the growth and productivity of the microalga, nutrient composition and light intensity were selected based on optimized conditions.

### Detection of intracellularly synthesized lipids by fluorescence microscopy

The total lipids synthesized within the cells were observed using a fluorescence microscope (Invitrogen EVOSTM FL; Thermo Fisher Scientific, Waltham, Massachusetts, USA) fitted with a fluorescence LED light cube (GFP_470/525 nm_).

The microalgal cells were dyed with neutral lipid staining dye BODIPY_493/503_ (Invitrogen) at a concentration of 0.1 mg/mL in DMSO. In the stationary phase, 1-mL culture samples were centrifuged and the resulting pellet was resuspended in deionized water (200 μL) and followed by the addition of 2 μL of the lipid staining dye BODIPY_493/503_. The samples were stored in the dark for 10 min to allow for dye penetration and binding to the lipid structures within the cells. Finally, a small amount of the stained cell suspension was kept on a glass slide (VWR, Stockholm, Sweden) and visualized by microscope.

### Cell dry weight and lipids measurement from the batch cultivations of *B. braunii*

During autotrophic cultivation, the OD at 680 and 720 nm was assessed using a built-in multicultivator for each tube, with an optical path length of 27 mm. The OD was monitored at 10-min intervals. Biomass from each tube was collected through centrifugation and subsequently subjected to freeze-drying. The dry biomass (g/L) was quantified gravimetrically. For autotrophic cultivations in Erlenmeyer flasks, flat-panel photobioreactor, and open raceway pond, samples were taken from the cultures at 24-h intervals to assess cell growth. This resulted in a total of 16 samples collected over a 15-day period, starting from day 0. To harvest the cells from active cultures, samples (10 mL) were centrifuged at 10,000 rpm for 10 min, freeze-dried until a constant dry weight was achieved, and estimated through gravimetric measurement. To extract the lipids, the biomass was ground into a fine powder using a mill and pestle, and then mixed with chloroform:methanol solution in 2:1 (v/v) ratio. The mixture underwent continuous shaking for 2 h to ensure thorough homogenization. After adding half of its amount of distilled water, the slurry underwent centrifugation to separate the phases. The organic solvent layer, which contained the lipids, was then carefully transferred to glass vials that were previously weighed. Subsequently, the solvent was evaporated to leave behind the isolated lipids. The remaining lipids were weighed to estimate their content as a percentage (wt/wt) of the total lipid concentration relative to the cell dry weight.

### Analysis of hydrocarbons profile by GCMS

Hydrocarbons were extracted from freeze-dried microalgal biomass as detailed by Cheng et al. [4]. In brief, *n*-hexane was used to pulverize 50 mg of freeze-dried biomass, and the resultant extraction was centrifuged. The described procedure was replicated three times, and the resulting supernatant from each repetition was pooled and collected in pre-weighed glass vials. Crude hydrocarbons were determined gravimetrically and purified with column chromatography on silica gel using *n*-hexane as solvent, whereas remaining lipids were removed by chloroform and methanol. Purified hydrocarbons were analyzed by GC–MS (Clarus 690 MS coupled to Clarus SQ8 GC instrument; PerkinElmer, Waltham, MA, USA) equipped with a capillary column Elite 5MS, Cat. # N9316282; (PerkinElmer, Waltham, MA, USA).

### Characterization of fatty acids profile from extracted lipids

To assess the fatty acid profile, lipid transesterification was performed using the protocol provided by Wychen et al. with a few modifications [23]. The lipids were suspended in a chloroform:methanol mixture (2:1, v/v) and combined with acid catalyst 0.6 M HCl in methanol. After preparing the mixture, it was transferred into ace pressure tubes, which were subsequently positioned in a water bath at 85 °C for an hour. Then, using *n*-hexane, fatty acid methyl esters (FAME) were extracted and prepared for GC–MS estimation equipped with a capillary column (Elite -FFAP; PerkinElmer, Waltham, MA, USA). Fatty acid profiles were analyzed as detailed in section "Analysis of hydrocarbons profile by GCMS". Temperature settings during GC–MS were as described previously [5].

### Thin-layer chromatography (TLC) of crude hydrocarbons and lipids

TLC was used to analyze the hydrocarbons in the total lipids on silica gel 60 plates [24]. The mobile phase was composed of an 85:15:1 (v/v/v) ratio of *n*-hexane, diethyl ether, and acetic acid. To visualize the separated bands, a solution of methanolic MnCl_2_ was sprayed on the plate. This solution was prepared by combining 0.32 g MnCl_2_·4H_2_O, 30 mL water, 30 mL methanol, and 4 mL concentrated H_2_SO_4_. The plate was then placed in a hot-air oven at 125 °C for 5 min to sear it. The band corresponding to triacylglycerols (TAG) and squalene was revealed by comparison with pure squalene (≥ 98%, liquid, S3626; Sigma-Aldrich) and using triolein as the standard.

### Assessment of protein and pigments from photoautotrophically grown microalgae

Pigments, including chlorophyll a, b and carotenoids, were measured in autotrophically grown microalgae cultivated in the three types of vessels (as detailed in sections. "Batch cultivation of Botryococcus braunii photoautotrophically" and "Cultivation in flasks and a flat-panel photobioreactor"). During the early stationary phase, the cells were collected by centrifugation at 10,000 rpm for 10 min, washed with distilled water, and combined with an equivalent volume of methanol. This mixture was incubated at 45 ℃ with shaking for 24 h. After the cells were centrifuged out of the supernatant, the absorbance (A) of the supernatant was assessed at 665, 652, and 470 nm using a UV–vis spectrophotometer (Spectra Max M2). The obtained pigments (Chl a, Chl b and carotenoids) were quantified (in μg/mL) by equations mentioned below [25]:

$$\mathrm{Chl a}=16.72\times {A}_{665}-9.16\times {A}_{652},$$

$$\mathrm{Chl b}=34.09\times {A}_{652} -15.28\times {A}_{665},$$

$${\text{Carotenoids}}={}^{(1000 {A}_{470}-1.63\mathrm{ Chl a}-104.9\mathrm{ Chl b})}/{}_{221}.$$

In the early stationary phase, the total N content of the microalgae was determined using the Kjeldahl method. Freeze-dried microalgal samples (1 g) were digested with a DK6 heating digester and distilled using a Kjeldahl distillation unit; UDK139 (VELP Scientifica, Usmate, Italy). The resulting distillate was titrated with 0.1 N HCl. The assessment was carried out in duplicates, following guidelines outlined in the 978.04 AOAC method. The protein content was determined by applying the widely accepted nitrogen-to-protein conversion factor of 6.25.

### Scanning electron microscopy (SEM) of freeze-dried biomass

Scanning electron microscopy (SEM) was conducted using the high-resolution Magellan 400 system (FEI Company, Eindhoven, Netherlands), operating at an energy of 3.0 kV. Before analysis, samples were coated with a fine layer of tungsten.

### Thermal analysis of green, white, and de-oiled biomass

TGA was performed with 2–4 mg of biomass using a PerkinElmer TGA 8000 apparatus under N_2_ heated at 10 °C/min, where the spot of the baseline mass and the tangent of the mass vs temperature curve was calculated to determine the decomposition start temperature by using Pyris software from PerkinElmer, USA. In differential scanning calorimetry, 2–5 mg samples were placed in aluminum pans within a PerkinElmer DSC 6000 device. Data were collected during both cooling and heating at a 5 °C/min scan rate under a continuous 20 mL/min flow of dry N2 gas. To determine the glass transition temperature (Tg), the midpoint of the initial S-shaped transition slope was identified. Accurate Tg determination was aided by identifying the onset of the transition using Pyris software.

### Pyrolysis of whole, white, and de-oiled biomass

Pyrolysis GC–MS analysis was conducted using a Single shot PY-3030S pyrolyzer at 600 °C (Shimadzu, Kyoto, Japan), coupled with a PerkinElmer Clarus GC–MS 690/SQ8T system. The instrument was equipped with a Restek RTX-1701 column (60 m × 0.25 mm, 0.25 μm), and a quadrupole mass spectrometer detector utilizing electrospray ionization at 70 eV and an ion source temperature of 240 °C. An injection temperature of 280 °C and a split ratio of 1:10 were employed. The oven temperature initially held at 40 °C for 1 min, was then ramped at a rate of 8 °C/min to 280 °C and maintained for 45 min. The carrier gas, helium (He), flowed at 1 mL/min. Mass spectra were recorded in the range of 50–600 m/z.

## Results and discussion

### Impact of medium composition on the production of lipids and hydrocarbons by *B. braunii* SAG 807-1 cultivated in a mini-photobioreactor

**Fig. 2:**
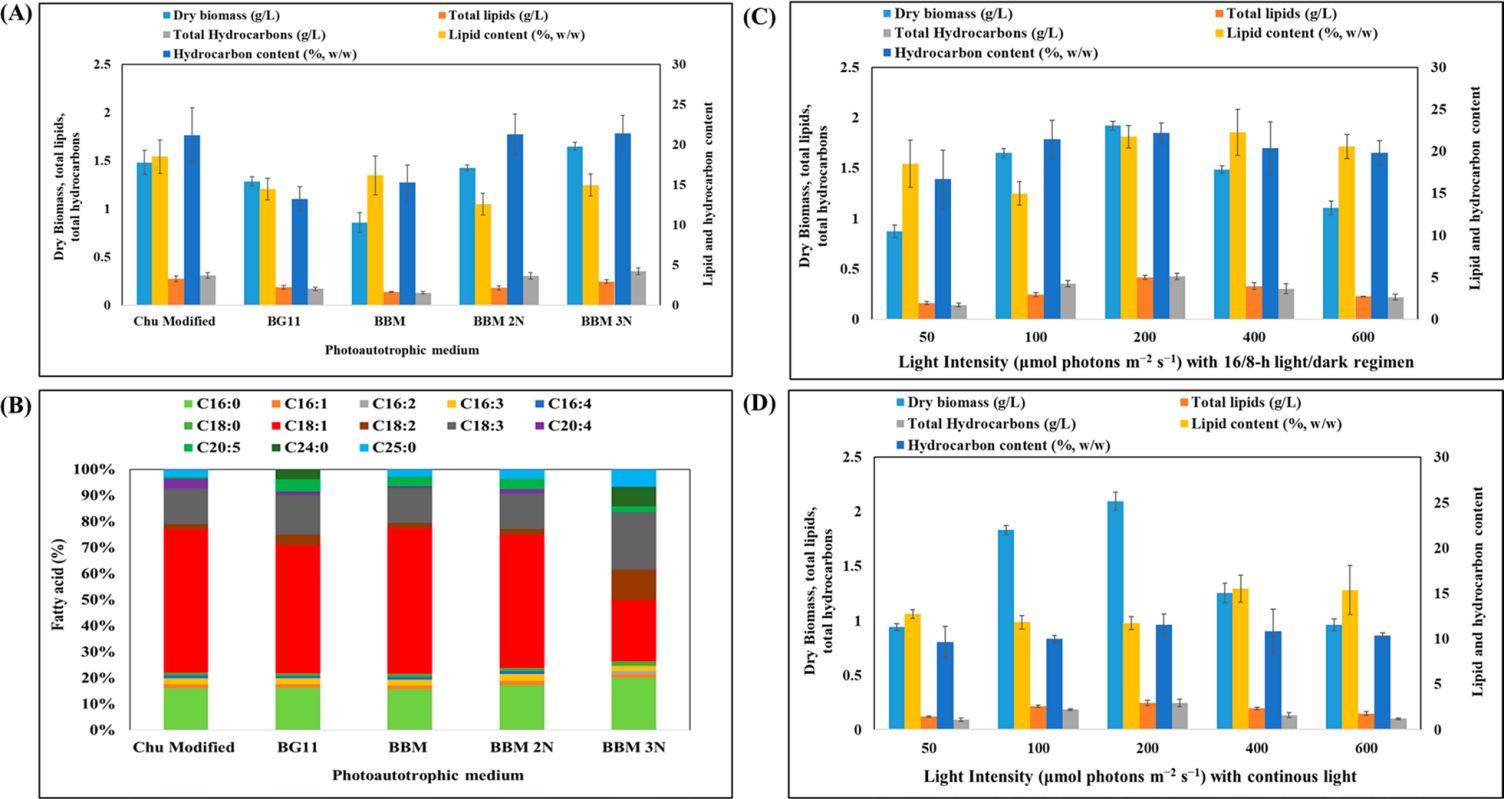
**A** Batch cultivation of *B. braunii* for cell dry biomass (g/L), total lipid (g/L), and total hydrocarbon (g/L) production in an airlift photobioreactor under photoautotrophic conditions with light intensity of 100 μmol/m^2^/s and a 16/8-h light/dark regimen. **B** Effect of varying the photoautotrophic medium on the fatty acid profile (%, total lipids) of *B. braunii*. Data represent the average of three individual GC–MS experiments. Effect of light intensity on lipid and hydrocarbon production by *B. braunii* cultivated in an airlift photobioreactor under a 16 h/8 h light–dark regimen (**C**) and continuous mode (**D**)

*Botryococcus braunii* is acknowledged for producing large amounts of extracellular hydrocarbons and intracellular lipids. Optimization of nutrients present in cultivation media is a crucial step for increasing microalgal biomass yield and metabolites production [10, 26]. Several media, including Chu 13, BBM and BG-11, are suitable for lipid and hydrocarbon production by *B. braunii* [27]. Lipid production from oleaginous microalgae is influenced by pH, light exposure and intensity, aeration rate, temperature, and nutrient absorption, as well as growth medium constitutes, such as carbon, nitrogen, and phosphorous in a specific ratios [28]. Stress conditions such as nutrient starvation are employed to induce lipid biosynthesis, while no such role has been associated with hydrocarbon production in this microalga [27, 29, 30]. Understanding how stress factors affect hydrocarbon output could further improve microalgae-based processes. In this study, biomass, lipids, and hydrocarbon production were optimized by cultivating *B. braunii* SAG in media with different compositions, such as modified Chu 13, BBM, and BG-11, in an airlift photobioreactor under photoautotrophic conditions with 100 μmol/m^2^/s light intensity and a 16/8-h light/dark regimen. BBM enriched with various amounts of nitrogen (250–750 mg/L NaNO_3_), was used as basal medium. The same was done with BG-11. The use of BBM as a cultivation medium yielded 0.86 ± 0.10 g/L cell dry weight, with 0.14 ± 0.004 g/L lipids and 0.13 ± 0.01 g/L crude hydrocarbons. These values increased to 1.65 ± 0.05 g/L cell biomass, 0.25 ± 0.02 g/L lipids, along with 0.35 ± 0.03 g/L crude hydrocarbons as the nitrogen content in BBM augmented from 250 mg/L to 750 mg/L (Fig. 2). These results were comparable with those obtained by Velichkova et al. (2012), whereby *B. braunii* showed high optical density (2.23) and cell dry weight (1.84 g/L) when cultivated in BBM 3N compared to BBM and suggested that growth improved with nitrate enrichment [31].

Lipid content on a dry biomass basis decreased from 16.18 ± 2.41% to 14.96 ± 1.39% when nitrogen augmented from 250 mg/L to 750 mg/L (Fig. 2A). These findings supported the idea that lipids accumulate in oleaginous microalgae under nitrogen limited condition. They were also similar to those obtained by Choi et al. (2011) with *B. braunii* UTEX 572 in Chu-13 medium including 0.04, 0.37, and 3.66 mM nitrate. Under nitrogen limitation (0.04 mM), the lipid content was almost two times higher than at a higher nitrogen content (3.66 mM); although the highest lipid concentration (0.19 g/L) was obtained with 0.37 mM nitrate [32]. In the present study, BG-11 medium contained nearly twice as much nitrogen as BBM 3N (1500 mg/L); yet, the attained cell dry weight (1.28 g/L) was only marginally higher than that obtained with BBM 2N and 3N or modified Chu medium. In contrast, total lipids were lower (0.18 ± 0.20 g/L) with BG-11 than with BBM 3N (0.24 ± 0.02 g/L) and also with modified Chu medium (0.27 ± 0.028 g/L) (Fig. 1). Velichkova et al. proposed that *B. braunii* experienced enhanced growth in a BBM 3N environment, which promoted higher biomass and lipid percentages [31]. However, Fang et al. suggested that, while an optimal nitrogen concentration was necessary for growth, a nitrogen-deficient environment could stimulate lipid synthesis in *B. braunii* [33].

The choice of medium was resulted in varying *B. braunii* biomass and lipid productivity (Table 1). The highest biomass productivity (110.00 ± 2.88 mg/L/day) was accomplished with BBM 3N, followed by modified Chu medium (98.88 mg/L/day). Such value was higher than those reported previously with the same strain: 75 mg/L/day [34] and 60 mg/L/day [35]. In contrast, the highest lipid productivity was recorded with the modified Chu medium (18.22 ± 1.91 mg/L/day), followed by BBM 3N (16.44 ± 1.37 mg/L/day). Therefore, the quantity of nitrogen in the medium had a direct impact on both biomass and lipid productivity. When the BBM medium was used and the nitrogen content was increased, a corresponding boost in both biomass and lipid productivity was observed. Higher nitrogen content leads to the synthesis of a larger amount of photosynthetic pigments. da Silva et al. conducted a study, which revealed that a decrease in nitrogen source resulted in reduced growth, chlorophyll content, and biomass. Additionally, nitrogen deficiency resulted in a quick drop in nitrogen-containing substances, such as photosynthetic pigments, which eventually led to a full loss of photosynthetic effectiveness [36]. However, when the BG-11 medium, which had almost double the nitrogen content compared to BBM 3N, was utilized in the present study, a decline in both biomass and lipid productivity was observed. Hence, both these parameters appear to depend also on medium components other than nitrogen. In fact, several studies have demonstrated the influence of various media components on the growth and lipid synthesis in oleaginous microorganisms. Besides the amount, the form in which nitrogen is present also affects biomass and lipid production in *B. braunii* SAG 807-1 [27]. While nitrates are preferred, when both ammonium and nitrate are present in the culture medium, microalgal cells would take up ammonium first [37]. When trying five different nitrogen sources, Cheng et al. showed that KNO_3_ and NaNO_3_ were best for biomass and hydrocarbon assimilation by *B. braunii* SAG 807-1. Additionally, even though KNO_3_ is a component in the Chu 13 medium, NaNO_3_ is less costly [38], which can be part of the benefits in industrial processes. On this basis, we selected NaNO_3_ to evaluate biomass, lipid, and hydrocarbon production by this alga.

**Table 1:** Estimation of biomass, lipid, and hydrocarbon productivity by *B. braunii* cultivated in photoautotrophic mode with a light intensity of 100 μmol/m^2^/s and a 16-h light and 8-h dark regimen

Photoautotrophic medium	Biomass productivity (mg/L/day)	Lipid productivity (mg/L/day)	Hydrocarbon productivity (mg/L/day)
Modified Chu	98.88 ± 8.31	18.22 ± 1.91	20.67 ± 1.96
BG-11	85.78 ± 2.99	12.44 ± 1.36	11.33 ± 1.09
BBM	57.33 ± 6.82	9.11 ± 0.31	8.67 ± 0.94
BBM 2N	95.11 ± 1.91	12.00 ± 1.44	20.22 ± 2.20
BBM 3N	110.00 ± 2.88	16.44 ± 1.37	23.56 ± 2.19

In a study by Xin et al. on the oleaginous microalga *Scenedesmus* sp., nitrogen concentration, carbon source, phosphorus, and trace elements all significantly influenced biomass and lipid accumulation [39]. Optimizing these components resulted in improved lipid productivity. In another study by Ratledge and Wynn, nitrogen limitation, carbon-to-nitrogen ratio, phosphorus limitation, and other nutrient deficiencies also played critical roles in lipid production by oleaginous microorganisms. In particular, the authors emphasized the importance of balancing nutrient availability to achieve higher lipid yields [40].

Any biologically derived fats can be transesterified to produce FAME, also known as biodiesel. Biodiesel quality is entirely dependent on the lipid profile of feedstocks. Microalgae feature among the best feedstocks for the synthesis of abundant neutral fats, such as C16–C18 fatty acids. The prevalent fatty acid profile in biodiesel consisted of palmitic (C16:0), stearic (C18:0), oleic (C18:1), and linolenic acid (C18:3) [41]. Myristic acid (C14:0), stearic acid, palmitoleic acid (C16:1), palmitic acid, oleic acid, and linoleic acid (C18:2) are combined within TAGs and phospholipids during transesterification, resulting in the formation of FAME. The specific fatty acid profile and its content, however, varying on the strains of oleaginous microalgae and the culture conditions employed. *B. braunii* cultivated on modified Chu medium contained abundant oleic acid (55.01%), followed by palmitic acid (16.07%) and linolenic acid (13.67%) (Fig. 2B). These three fatty acids covered 85% of total fatty acids, with the rest accounted by C16:1, C16:3, C16:4, C18:0, C18:2, C20:4, and C20:5. The fatty acid profile was comparable between *B. braunii* grown on BG-11 and BBM; the only significant variation was the level of eicosapentaenoic acid (C20:5), which was 4.89% with BG-11 and 3.69% with BBM, plus the presence of C24:0 (3.65%) on BG-11. A dramatic drop in oleic acid (23.57%) was observed when shifting from BBM to BBM 3N (Fig. 2B). The observed increment in oleic acid under nitrogen-limited conditions was in agreement with previous results with other *B. braunii* strains cultivated in suspension [42]. *B. braunii*, FACHB 357 contained mostly oleic acid (52.25%), linolenic acid (15.81%), and palmitic acid (11.32%). According to Cheng et al. (2013), oleic acid is most abundant while microalgae are cultured either under sufficient or deficient nitrogen [42].

### Impact of light intensity on lipid and hydrocarbon production under a 16/8-h light–dark regimen and continuous mode

Light plays a central role in the growth of microalgae, as it affects their growth rate, reproduction, cell morphology, and metabolism. Quality and intensity of light are essential for optimal productivity of microalgal cultures. The main mechanism by which microalgae transform light energy into chemical energy and biomass is photosynthesis. Adequate light intensity and duration ensure a sufficient energy supply for photosynthesis, thereby promoting cell division and reproduction. Distinct algal species have evolved under different environmental conditions, and their growth patterns and physiological responses can be influenced by the duration of light and dark periods. In this study, *B. braunii* was cultivated under different light intensities (50–600 μmol photons/m^2^/s, continuous mode, and 16/8-h light–dark regimen (Fig. 2C). Biomass exhibited a positive correlation with light intensity under a 16/8-h light/dark regimen, increasing from 0.87 ± 0.06 g/L at 50 μmol photons/m^2^/s to 1.65 ± 0.04 g/L at 100 μmol photons/m^2^/s, and peaking at 1.92 ± 0.05 g/L at 200 μmol photons/m^2^/s. Any further increments in light intensity failed to improve biomass yields and, in fact, the latter dropped to 1.49 ± 0.04 g/L at 400 μmol photons/m^2^/s (Fig. 2C). Lipid content exhibited a similar consistent pattern, going from 0.16 ± 0.02 g/L at 50 μmol photons/m^2^/s to 0.42 ± 0.02 g/L at 200 μmol photons/m^2^/s. However, beyond this point, the lipid concentration declined to 0.33 ± 0.03 g/L. A similar trend in lipid concentration was observed when expressing lipid concentration in terms of cell dry weight. The highest hydrocarbon concentration (0.42 ± 0.03 g/L) was achieved at 200 μmol photons/m^2^/s, failing to increase any further at higher light intensities (Fig. 2C).

In the subsequent set of experiments, microalgae were cultivated under continuous light mode, using similar light intensities as in the previous set (Fig. 2D). Under these conditions, significantly more biomass was obtained compared to light and dark conditions. At an irradiance of 200 μmol photons/m2/s, the highest biomass production was observed, reaching 2.09 ± 0.08 g/L. However, increasing the light intensity beyond this point did not result in any further improvements in biomass yield (Fig. 2D). According to Ruangsomboon, cultivation of *B. braunii* KMITL2 under a continuous light mode resulted in 1.91 ± 0.24 g/L biomass, which was four times more than under a 12/12-h light–dark regimen [43]. Changing the light cycle to 16/8-h and 14/10-h did not significantly affect biomass yields, while a drop in biomass was observed at a 12/12-h light–dark cycle. Biomass augmented with an increase in light intensity from 0.3 μE/m^2^/s to 87.5 μE/m^2^/s, followed by a drop at 200 μE/m^2^/s, and then an increase again at 538 μE/m^2^/s. In line with our study, Ruangsomboon found that the highest lipid yield (0.59 g/L) was obtained under a 16/8-h light cycle, while the lowest yield was observed under a 24/0-h light cycle. Qin and Li suggested that growth of *B. braunii* CHN 357 was optimal in SE medium containing 0–0.15 M NaCl, at 23 °C, with 30–60 W/m^2^, and a 12/12-h photoperiod, although it could survive at up to 200 W/m^2^ [44]. Cheng et al. proposed a distinct pattern for the association between light intensity and biomass productivity in an attached photobioreactor of *B. braunii* FACHB 357 [4]. At an initial light intensity of 10–60 μmol/m^2^/s, there was a linear four-fold increase in biomass productivity to 4.42 g/m^2^/day. However, as light intensity was further increased, biomass productivity slowed down, eventually reaching a plateau at 7.42 g/m^2^*/*day when light intensity exceeded 150 μmol/m^2^/s. These findings indicate that, beyond a certain threshold, increasing the light intensity does not result in a significant increase in biomass productivity [42]. According to Khichi et al., the design of algal photobioreactors needs to consider the self-shading phenomenon, which arises when microalgal cells diminish the extent of light penetration within the system. A high light intensity of 800 μmol photons/m^2^/s resulted in the highest biomass production (1.8 g/L) [45]. However, the maximum lipid content (27.37%) was reported at half of that light intensity, specifically 450 μmol photons/m^2^/s [45]. Zang and Kojima suggested that when the cell density was low and an adequate supply of light was available for photosynthesis, colony size tended to increase. This is because a low cell concentration allows for sufficient light to penetrate the colony, facilitating photosynthesis and supporting cell growth and colony expansion. As the cell concentration increases over time, mutual shading starts to occur within the colony, with cells in the interior blocking or reducing the amount of light reaching those in the outer layers. As a result, average light intensity within the photobioreactor decreases [46]. *Botryococcus* sp. MCC 32 synthesized a higher amount of lipids when cultivated in BG-11 medium at a light intensity of 95 μE/m^2^/s under a 16/8-h light/dark cycle than those grown in BBM and TAP media [47].

### Effect of various cultivation strategies on the production of lipids and hydrocarbons

**Fig. 3:**
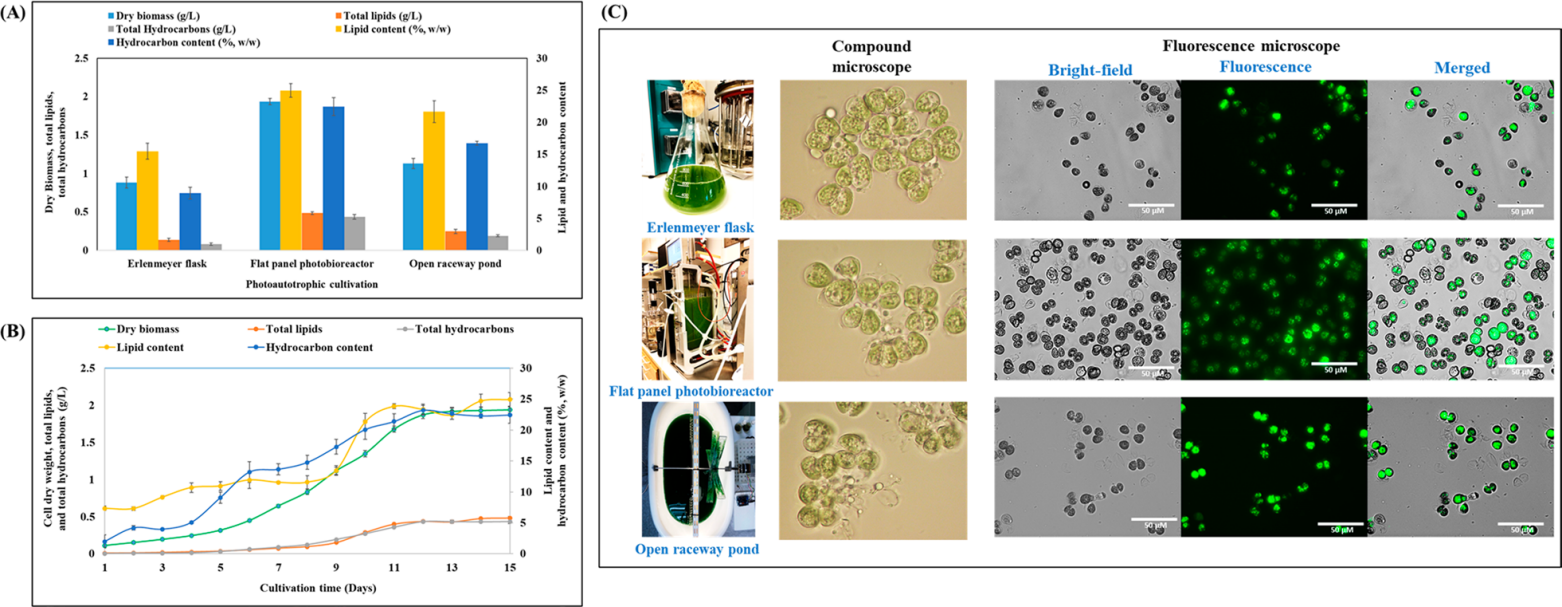
**A** Effect of various cultivation strategies on dry biomass, lipids, and hydrocarbon production by *B. braunii* cultivated in BBM 3N medium with light intensity of 200 μmol photons/m^2^/s and 16/8-h light/dark regimen. **B** Time-course experiment measuring cell dry weight, total lipid, and hydrocarbons produced by *B. braunii* during photoautotrophic cultivation in a flat-panel photobioreactor with light intensity of 200 μmol photons/m^2^/s and 16/8-h light/dark regimen. **C** Images of *B. braunii* cultivated in an Erlenmeyer flask, flat-panel photobioreactor, and open raceway pond, with light intensity of 200 μmol photons/m^2^/s and 16/8-h light/dark regimen (left panels). Fluorescence microscopy images of *B. braunii* cultures stained with BODIPY dye to visualize lipid accumulation (right panels)

In this work, three distinct types of aqueous suspended culture systems, including Erlenmeyer flasks, a flat-panel bioreactor, and an open raceway pond, were examined for *B. braunii* biomass, lipids, and hydrocarbon production (Fig. 3A). Based on accrued evidence, the most suitable light intensity (200 µmol/m^2^/s), photoperiod (16/8-h light–dark regimen), medium composition (BBM 3N), and temperature (25 °C) were applied to all three culture systems. The flat-panel bioreactor yielded the highest cell dry weight (1.93 ± 0.04 g/L), followed by the open raceway pond (1.13 ± 0.06 g/L) Erlenmeyer flasks (0.88 ± 0.07 g/L). The high biomass obtained using the flat-panel bioreactor could be attributed to controlled temperature and aeration. The lipid concentration was 0.48 ± 0.02 g/L (Erlenmeyer flasks), 0.24 ± 0.03 g/L (flat-panel bioreactor), and 0.13 ± 0.02 g/L (open raceway pond), equivalent to a lipid content of 24.95 ± 1.08%, 21.63 ± 1.70%, and 15.46 ± 1.25% based on cell dry weight. Results obtained with the flat-panel bioreactor were even better than those recorded with an airlift mini-photobioreactor (60 mL), whereby dry biomass, total lipid concentration, and lipid content were 1.65 g/L, 0.35 g/L, and 14.96%, respectively (Fig. 2A). Unique carbon partitioning during photosynthesis has a significant impact on the speed of microalgal growth. In contrast to most plants and microalgae, whereby 85% of the absorbed carbon goes into biomass synthesis, only 45% of the assimilated carbon is directed towards growth in *B. braunii* [48]. According to Banerjee et al., *B. braunii* cultures likely respond to nitrogen starvation by breaking down nitrogen-containing macromolecules and accumulating carbon reserve compounds, such as polysaccharides and fats [10]. This indicates that, when nitrogen is limited, the organism tends to store carbon-based compounds. In addition, Xu et al. observed a down-regulation of photosynthetic activity in response to nitrogen deprivation. This finding suggests that lipid accumulation in *B. braunii* is unlikely to be solely due to the synthesis of lipids from extracellular carbon sources. Instead, it might result from a destructive process, in which the organism breaks down internal nitrogen-containing compounds to generate carbon-rich compounds such as lipids [49].

Maximum biomass productivity (129.11 ± 2.74 mg/L/day) was attained with a flat-panel bioreactor, followed by cultivation in an open raceway pond (75.33 ± 4.32 mg/L/day) and Erlenmeyer flasks (64.00 ± 4.65 mg/L/day) (Table 2). Time-course experiments were conducted on *B. braunii* cultivated in a flat-panel photobioreactor under 200 μmol photons/m^2^/s and a 16/8-h light/dark cycle. The production of cell dry biomass, total lipids, and hydrocarbons was recorded during a 15-day period (Fig. 3B). During the initial lag phase, specifically on the 6th day, biomass remained below 0.5 g/L, along with low total lipid (0.053 g/L) and hydrocarbon (0.058 g/L) levels. Biomass, lipids, and hydrocarbon synthesis peaked between the 7th and 13th day. Subsequently, lipid content presented only a slight increase, from 0.43 g/L (22.43%) to 0.48 g/L (24.96%). This indicates that growth rate and lipid production reached a plateau. Finally, the highest biomass (1.93 g/L) was achieved on the 15th day, along with 0.48 g/L total lipids along with 0.43 g/L hydrocarbons.

**Table 2:** Estimation of biomass, lipid, and hydrocarbon productivity (mg/L/day) by *B. braunii* cultivated under photoautotrophic conditions in Erlenmeyer flasks, a flat-panel photobioreactor, and an open raceway pond, under light intensity of 200 μmol photons/m^2^/s and 16/8-h light/dark regimen

Photoautotrophic medium	Biomass productivity (mg/L/day)	Lipid productivity (mg/L/day)	Hydrocarbon productivity (mg/L/day)
Erlenmeyer flask	64.00 ± 4.65	9.11 ± 1.30	5.27 ± 0.94
Flat-panel photobioreactor	129.11 ± 2.74	32.21 ± 1.31	28.98 ± 2.08
Open raceway pond	75.33 ± 4.32	16.33 ± 1.89	12.60 ± 0.93

These findings confirm earlier results, which revealed the slower growth of *B. braunii* compared to other microalgae. However, this slower growth rate is compensated by a significantly higher lipid content [50]. Specifically, in the case of *B. braunii* SCCAP 1761, lipids constitute as much as 80% of its dry weight. Meanwhile, various other strains of microalgae have demonstrated an average lipid yield surpassing 57% of dry weight [50]. However, contrasting these findings, certain green freshwater microalgae like *Chlorella* or *Scenedesmus* tend to accumulate up to 43% of total lipids, predominantly in the form of TAGs, under conditions of stress or limited nitrogen availability [51]. Hydrocarbon production is also linked to *B. braunii* growth [52], highlighting the potential of *B. braunii* as a microalga with high lipid content and hydrocarbon production, despite its slower growth rate. This characteristic makes it an interesting candidate for various applications, particularly in the field of biofuel production or other lipid-based industries [52].

Lipid production is common to all microalgae; however, *B. braunii* has the added capacity of storing these lipids outside the cell, in an extracellular matrix [53, 54]. This capability facilitates downstream processing by enabling lipid extraction without cell death, maintaining a live culture throughout the harvesting phase, significantly lowering the nutritional requirements of the culture, and reducing the high energy intake required for dewatering microalgae during in situ lipid extraction [55, 56].

Morphological analysis using a compound microscope revealed that flask cultivation resulted in a higher number of intact cells compared to growth in a flat-panel photobioreactor or an open raceway pond (Fig. 3C). Lipid synthesis was assessed by staining the cells with the BODIPY fluorescent dye, which labels specifically neutral lipids. Live fluorescence microscopy images revealed numerous small lipid droplets in *B. braunii*, irrespective of cultivation method. The latter had no bearing also on lipid droplet characteristics and cell size (Fig. 3C). Hence, all three cultivation methods supported lipid synthesis; however, flask cultivation resulted in more intact cells compared to the other two methods.

**Fig. 4:**
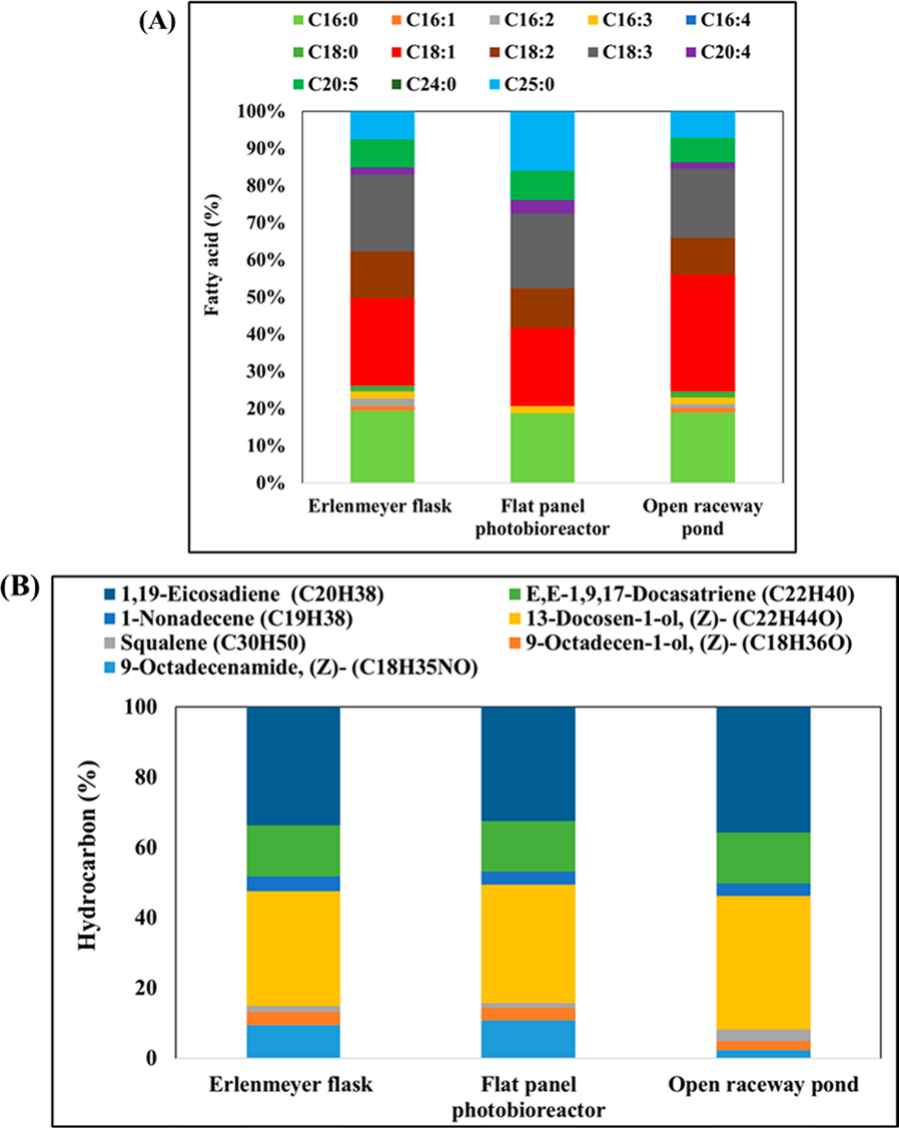
**A** Fatty acid profile (% of total lipids) of *B. braunii* cultivated in an Erlenmeyer flask, flat-panel photobioreactor, and open raceway pond, under light intensity of 200 μmol/m^2^/s with a 16/8-h light/dark regimen. **B** Production of hydrocarbons (%) by *B. braunii* cultivated under photoautotrophic conditions in Erlenmeyer flasks, flat-panel photobioreactor, and open raceway pond. Data represent the average of three individual GC–MS experiments

The fatty acid profile of *B. braunii* cultivated in the three different modes under 200 μmol photons/m^2^/s with a 16/8-h light/dark regimen was evaluated (Fig. 4A). The most prevalent fatty acids in Erlenmeyer flasks cultivations were C18:1 (23.49%), C18:3 (20.69%), and C16:0 (19.39%), followed by C18:2 (12.63%), C20:5 (7.60%), and C25:0 (7.50%). The remaining fatty acids accounted for approximately 5.31% of the total. In contrast, cultivation in a flat-panel photobioreactor yielded a slightly lower percentage of C18:1 (20.99%) but a larger amount of C25:0 (16.03%) compared to other methods, with C18:2 (10.01%) and C18:3 (20.79%) accounting for significant proportions. Finally, cultivation in an open raceway pond resulted in a similar fatty acid profile as with Erlenmeyer flasks, except for a slightly higher proportion of C18:1 (31.43%) and a decrease in C18:2 (9.89%) and C18:3 (18.60%). These findings indicate that the fatty acid composition of *B. braunii* can vary depending on the cultivation conditions and vessel type. The Erlenmeyer flask culture exhibited a higher proportion of certain fatty acids, while the flat-panel photobioreactor and open raceway pond cultures showed some variations in C18:1, C18:2, and C18:3. Ranga Rao assessed the growth dynamics, hydrocarbon production, and fatty acid profiles of *B. braunii* strains LB-572 and N-836 grown in raceway and circular ponds under outdoor conditions. After 18 days, cultures from both pond types showed higher biomass yield and hydrocarbon content. Lipids, representing approximately 24% (wt/wt), were dominated by palmitic and oleic acids [57].

Different cultivation strategies resulted in varying amounts of hydrocarbons (Fig. 4B). The most abundant hydrocarbons were alkadienes, followed by 13-docosen-1-ol (Z) or erucyl alcohol as a lipophilic hydrocarbon and E,E-1,9,17-docasatriene. The level of 1,19-eicosadiene was uniform across all cultivation methods: 34.12% in Erlenmeyer flasks, 32.75% in a flat-panel photobioreactor, and 35.92% in an open raceway pond. A similar consistency was observed for 13-docosen-1-ol (Z) and E,E-1,9,17-docasatriene, which exhibited comparable ranges of 32.63% to 37.79% and 14.03% to 14.49%, respectively.

A US patent by Ippoliti et al. describes a novel type of polymer based on 13-docosen-1-ol, which comprises a lipophilic hydrocarbon segment and a biodegradable polymeric segment. The former allows the polymer to interact with hydrophobic materials; whereas the latter enables the polymer’s natural breakdown over time. The patent explains the synthesis and composition of these polymers, as well as their potential applications in drug delivery, coatings, and environmental remediation. The invention aims to provide versatile and environmentally friendly polymers with unique properties and applications [58].

*Botryococcus braunii* strain A produces nonisoprenoid hydrocarbons, specifically alkadienes and trienes. Alkadienes share a similar double bond location and stereochemistry with oleic acid (18:1, cis-ω9) [59]. Accordingly, oleic acid may serve as a direct precursor for the synthesis of *n*-alkadienes [60]. During periods of intensive hydrocarbon production, such as exponential and initial linear growth phases, the intracellular concentration of oleic acid remains relatively low [61]. However, during the deceleration phase, when hydrocarbon production decreases, oleic acid augments significantly.

The presence of a terminal double bond in the hydrocarbons and the inhibitory effect of dithioerythritol indicate that the biosynthesis of hydrocarbons (dienes) occurs through an elongation–decarboxylation pathway, rather than a head-to-head condensation pathway. In the elongation–decarboxylation mechanism, oleic acid functions as the direct precursor, undergoing successive elongation steps through the addition of C2 units derived from malonyl Co-A until the desired chain length is achieved. These elongated products with very long carbon chains are subsequently decarboxylated and released from the elongation–decarboxylation complex. The latter is not specific in *B. braunii* as it can accommodate both oleic acid and elaidic acid (18:1, trans-ω9) as substrates, leading to the production of both cis and trans dienes. Nitrogen limitation causes a decrease in C20–C30 hydrocarbons, as well as those longer than C30, but it favors those shorter than C20. Variability in the hydrocarbon profile might be due to the use of various strains or periods of growth [53, 57]. Moreover, divergent growth, hydrocarbon production, and fatty acid profiles of *B. braunii* strains in outdoor pond systems highlight the influence of cultivation duration, seasonality, and nutrient supplementation. Notably, the maximum biomass yield (2 g/L) and hydrocarbon content (28%) were observed during November to December [57]. During nitrogen-deficient conditions, the quantities of hydrocarbons (crude and pure) and non-polar lipids rose while polar lipids dropped; nevertheless, the total lipid content (polar + non-polar) altered only a little. Nitrogen deficiency boosted hydrocarbon biosynthesis [62], Hydrocarbon synthesis can be hindered in high nitrate conditions. Nitrogen deprivation can cause polar lipids to convert to non-polar lipids [63, 64], thereby overcoming lipid synthesis.

### Effect of cobalt stress on the production of hydrocarbons and lipids by *B. braunii* cultivated under photoautotrophic conditions in Erlenmeyer flasks, flat-panel photobioreactor, and open raceway pond

Cobalt is a trace metal that is vital in the metabolism of microalgae, where it forms the active site of vitamin B12. Cobalt stress can have both beneficial and negative impacts on microalgae hydrocarbon and lipid synthesis [65]. Cobalt stress, on the other hand, causes oxidative stress, which stimulates lipid metabolism and leads to increased lipid accumulation. In rare circumstances, hydrocarbon and lipid production might rise by up to 2–3 times. Excess cobalt can be harmful to cells, resulting in diminished growth and lipids accumulation. Furthermore, the response of microalgae to cobalt stress varies by strain, with certain strains showing no substantial increase in hydrocarbon or lipid synthesis under cobalt stress conditions [66]. While cobalt stress can accelerate the synthesis of hydrocarbons and lipids in specific microalgae strains, cobalt levels must be carefully managed to minimize toxicity and ensure optimum growth and lipid accumulation.

**Fig. 5:**
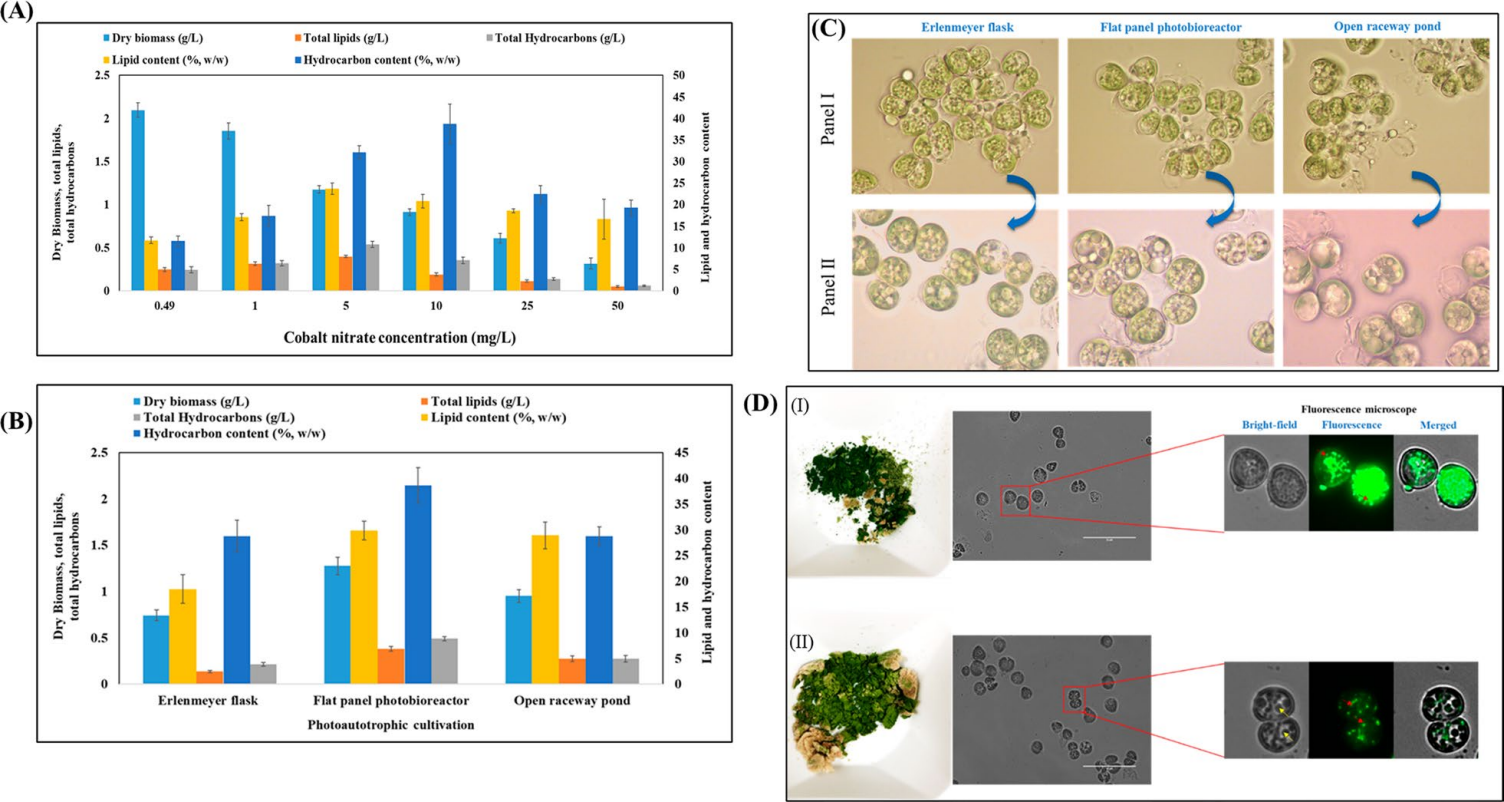
**A** Effect of cobalt nitrate concentration (0.49, 1, 5, 10, and 50 mg/L) on the production of biomass, lipids, and hydrocarbons by *B. braunii* cultivated in BBM 3N medium within a mini-photobioreactor. **B** Effect of elevated concentration cobalt nitrate on the production of biomass, lipids, and hydrocarbons by *B. braunii* cultivated under photoautotrophic conditions in Erlenmeyer flasks, flat-panel photobioreactor, and open raceway pond. **C** Light microscopy images showing the morphology of *B. braunii* cultivated in the presence of a normal cobalt concentration (0.49 mg/L; Panel I) and an elevated cobalt concentration (5 mg/L; Panel II). **D** Lipid accumulation in *B. braunii* cultivated in the presence of a normal cobalt concentration (0.49 mg/L) (I) and a high cobalt concentration (5 mg/L) (II). Representative bright-field and fluorescence images of cells stained with the lipid dye BODIPY_493-503_ are shown. Scale bars: 50 μm

In this study, we investigated the effect of different concentrations of cobalt nitrate (0.49, 1, 5, 10, and 50 mg/L) on the production of biomass, lipids, and hydrocarbons by *B. braunii* cultivated in BBM 3N within a mini-photobioreactor. A negative correlation was observed between cobalt nitrate concentration and biomass yield (Fig. 5A). The control group, with 0.49 mg/L cobalt nitrate, exhibited the maximum biomass production (2.09 ± 0.08 g/L), along with progressively higher cobalt nitrate concentrations resulting in decreasing biomass yields of 1.85 ± 0.08, 1.17 ± 0.08, 0.91 ± 0.08, 0.61 ± 0.08, and 0.32 ± 0.08 g/L.

On the contrary, lipid concentration and content increased from 0.25 ± 0.03 g/L to 0.40 ± 0.012 g/L and from 11.73 ± 0.72% to 23.69 ± 1.31%, respectively, when the cobalt nitrate concentration was raised from 0.49 mg/L to 5 mg/L in BBM 3N (Fig. 5A). A similar trend was observed for the hydrocarbon concentration, which also increased from 0.24 ± 0.03 g/L to 0.54 ± 0.04 g/L (Fig. 5A). However, any further increases in cobalt nitrate concentration failed to promote lipid formation and lowered the hydrocarbon output, which dropped to 0.06 ± 0.008 g/L at 50 mg/L cobalt nitrate.

To assess the effect of cultivation mode, *B. braunii* cultures were grown in Erlenmeyer flasks, airlift flat-panel photobioreactor, and open raceway pond at a cobalt nitrate concentration of 5 mg/L in BBM medium. Dry biomass yields were 0.74 ± 0.05 g/L (Erlenmeyer flasks), 1.27 ± 0.09 g/L (airlift flat-panel photobioreactor), and 0.95 ± 0.07 g/L (open raceway pond) (Fig. 5B). These values were slightly lower compared to those obtained without cobalt stress (0.49 mg/L cobalt nitrate). In the airlift flat-panel photobioreactor, the lipid concentration increased to 0.38 ± 0.026 g/L, while the hydrocarbon concentration reached 0.49 ± 0.02 g/L. In the open raceway pond, the corresponding values were 0.28 ± 0.03 g/L and 0.27 ± 0.03 g/L, which amounted to a lipid content of 28.92 ± 2.62% and a hydrocarbon content of 28.80 ± 1.79%. Without cobalt stress, the lipid content was 21.63 ± 1.69% and the hydrocarbon content was 16.7 ± 0.29%. Hence, lipid and hydrocarbon production increased under cobalt nitrate stress conditions (Fig. 5B). Table 3 lists the values of biomass, lipid, and hydrocarbon productivity (mg/L/day). Under cobalt stress, biomass productivity was slightly lower compared to non-stress conditions in all three cultivations. The flat-panel bioreactor yielded the highest productivity for biomass (85.11 ± 6.38 mg/L/day), lipids (25.35 ± 1.77 mg/L/day), and hydrocarbons (32.70 ± 1.53 mg/L/day). Interestingly, biomass productivity was lower under cobalt stress than reported previously without cobalt stress, but hydrocarbon productivity remained high. There is debate concerning cobalt function in photosynthesis. Its hazardous effect results from PSII activity suppression due to altering the secondary quinone electron acceptor QB site as it inhibits the PSII acceptor's reaction center or a component of it [67, 68]. Elevated concentrations of metals (particularly cobalt, exceeding 45 mg/L) in the growth medium have the potential to impact the functioning of the chloroplast's complex protein system. It is widely recognized that complex proteins in chloroplasts improve photosynthesis by augmenting their capacity to absorb light, ultimately stimulating the growth of microalgal cells [69]. The transcriptome of *B. braunii* over four time points during the cobalt treatment, as proposed by Cheng et al., showed that the most strongly expressed genes under high concentration were those related to fatty acid biosynthesis and metabolism, as well as oxidative phosphorylation; conversely, the most downregulated genes were related to carbohydrate metabolism, photosynthesis, and amino acid metabolism. The global gene transcript patterns indicated that different phenotypes under cobalt enrichment are likely caused by different expression dynamics [66].

**Table 3:** Effect of elevated concentration cobalt nitrate (5 mg/L) on the productivity (mg/L/day) of biomass, lipids, and hydrocarbons by *B. braunii* cultivated under photoautotrophic conditions in Erlenmeyer flasks, flat-panel photobioreactor, and open raceway pond

Photoautotrophic medium	Biomass productivity (mg/L/day)	Lipid productivity (mg/L/day)	Hydrocarbon productivity (mg/L/day)
Erlenmeyer flask	49.56 ± 3.68	9.05 ± 0.70	14.22 ± 1.36
Flat-panel photobioreactor	85.11 ± 6.38	25.35 ± 1.77	32.70 ± 1.53
Open raceway pond	63.56 ± 4.53	18.37 ± 2.08	18.37 ± 2.31

A morphological analysis of cells grown in the presence of cobalt was carried out (Fig. 5C). Cells grown under low cobalt conditions displayed small-sized lipid droplets (Panel I, Fig. 11); whereas those grown under elevated doses of cobalt exhibited numerous large droplets (Panel II, Fig. 5C), pointing to the accumulation of hydrocarbons. To confirm the intracellular synthesis of lipid droplets, we stained the growing cultures with BODIPY dye (Fig. 5D). Cells grown under an elevated cobalt concentration showed large droplets that were not tagged with the BODIPY dye (Fig. 5D,I), confirming the presence of intracellular hydrocarbons. In contrast, in cells grown under normal cobalt conditions, all droplets were tagged with the fluorescence dye (Fig. 5D,II), indicating an abundance of intracellular lipids. These observations provide visual evidence of the morphological differences in cells grown under high cobalt conditions, with prominent large hydrocarbon droplets, compared to those grown under normal cobalt conditions, where intracellular lipids are abundant.

Cheng et al. demonstrated that *B. braunii SAG* 807-1 was tolerant towards elevated cobalt doses when grown in a biofilm [13]. Here, we found that *B. braunii* could adapt to high cobalt concentrations also when grown in suspension. Interestingly, a low concentration of cobalt stimulated algal growth, while rather higher concentrations were lethal. The significance of cobalt in photosynthesis is still debated. Its toxicity is due mostly to the suppression of photosystem II (PSII). Cobalt can inhibit either the reaction center or a component of the PSII acceptor by modifying the secondary quinone electron acceptor QB site. This interference with PSII activity disrupts the photosynthetic process and can lead to the toxic effects of cobalt on the algae [67]. According to Cheng et al., colonial *Botryococcus* species may survive high levels of metallic cobalt. They found that after treatment with cobalt, the content of extracellular hydrocarbons in these species increased by approximately 20%. Interestingly, treatment with cobalt also caused changes in the structure of *Botryococcus*, particularly in extracellular hydrocarbons, which in turn induced changes in extracellular polysaccharides [13]. A subsequent study on the effects of high cobalt treatment on the growth and total carbohydrate content of *B. braunii* SAG 30.81 revealed that these parameters were not influenced as much by high cobalt as by its absence [65]. Under normal conditions, the extracellular polysaccharides of *B. braunii* SAG 30.81 consisted primarily of C5 and C6 sugars, as well as sulfate substitutions [65]. These findings highlight the ability of *Botryococcus* species to tolerate and respond to cobalt treatment, leading to changes in the composition of their extracellular hydrocarbons and polysaccharides.

**Fig. 6:**
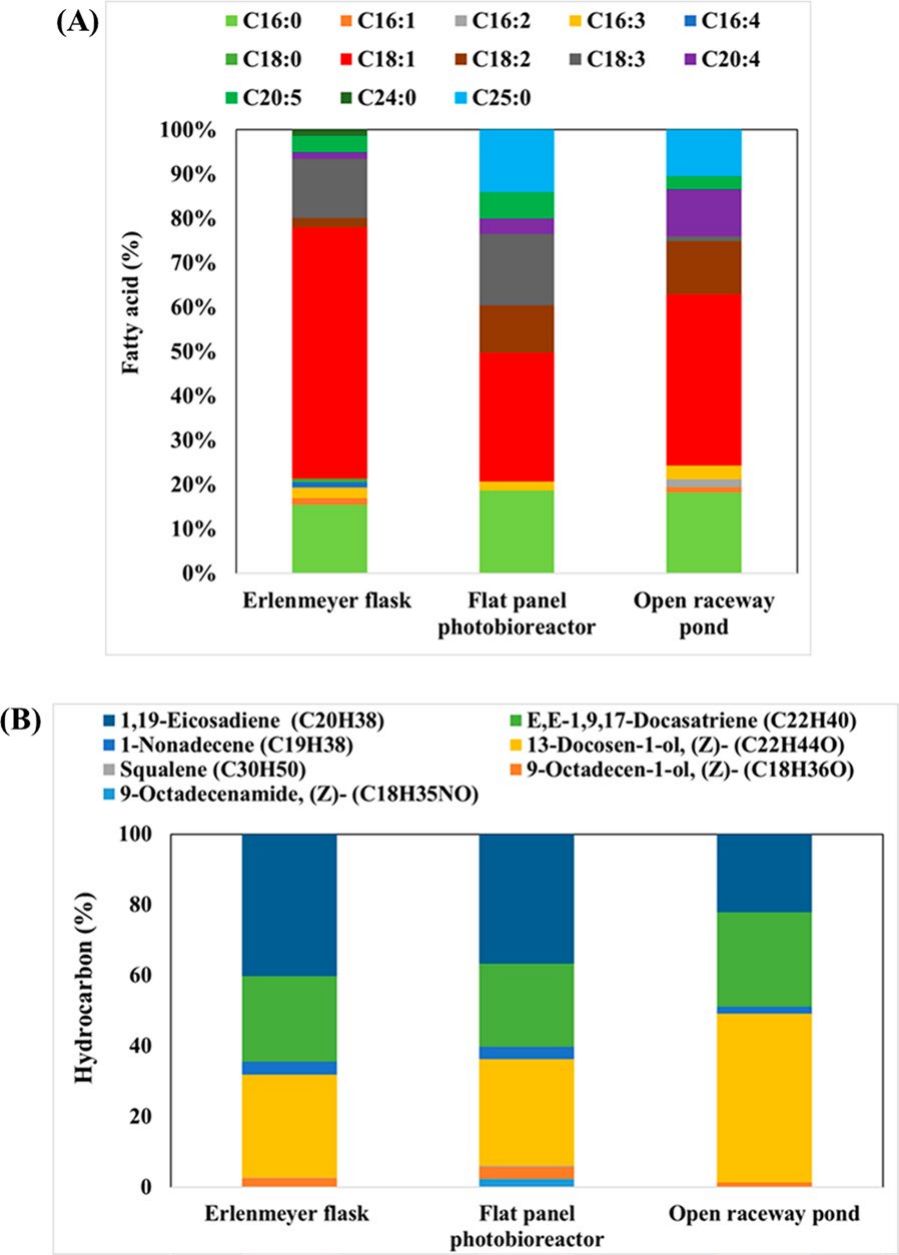
**A** Fatty acid profile of *B. braunii* cultivated in the presence of a high cobalt concentration (5 mg/L). Data represent the average of three individual GC–MS experiments. **B** Hydrocarbon profile of *B. braunii* cultivated in the presence of a high cobalt concentration (5 mg/L). Data represent the average of three individual GC–MS experiments

The fatty acid profile of cobalt-stressed cells varied with respect to the cultivation vessel (Fig. 6A). Erlenmeyer flasks yielded the highest proportion of C18:1 (56.69%), followed by C16:0 (15.49%) and C18:3 (13.28%). Interestingly, a new fatty acid, C24:0, was detected under these conditions, but was absent in non-stressed cultures. Conversely, C25:0 was absent under cobalt stress. In a flat-panel photobioreactor, the percentage of C18:1 (28.99%) was lower, yet still higher than in the absence of stress. Notably, the amount of C25:0 increased substantially (14.04%), while C18:2 and C18:3 accounted for 10.71% and 16.09%, respectively. Finally, the open raceway pond showed a similar fatty acid profile as the flat-panel photobioreactor, except for slightly more C18:1 (38.52%) and less C18:3 (1.14%). According to literature, the integrity of cell membrane in lag phase of growth is attributed to high amount of PUFA, whereas the early station phase of growth is characterized by the high levels of SFA (primarily C18:0) and MUFA (primarily C18:1). In a later stage of growth, Δ9 desaturase adds a double bond to transform C18:0 into C18:1 [70]. This requires a lot of oxygen, NADH, NADPH, and substrate, which ultimately stops the buildup of reactive oxygen species under stressful conditions [71]. By preventing the conversion of C18:0 to oleic acid (C18:1), the Δ9 desaturase inhibitor was used to demonstrate this oxidative mechanism [72].

*Botryococcus braunii* CCAP 807/2 has a unique composition, with palmitic acid, oleic acid, and octacosanoic acid (C28:1) accounting for up to 74% of all detected fatty acids in the polar fraction throughout all development stages (Phase II–IV) [73]. The proportion of oleic acid in the polar lipid fraction, on the other hand, fell during the period of culture, from 46.2 ± 1.2% in Phase II to 34.1 ± 4.3% in Phase IV. The decline in oleic acid concentration was much more pronounced in the non-polar lipid fraction, falling from 41.3 ± 4.6% in Phase II to as low as 7% in Phases III and IV [73]. These findings illustrate the dynamic nature of fatty acid composition in *B. braunii* CCAP 807/2 across several development stages, with variations in the proportions of oleic acid found in both the polar and non-polar lipid fractions.

Transcriptome analysis of *B. braunii* SAG 807–1 shed light on the early growth process and fatty acid biosynthesis [74]. During the initial growth stage (2 days), four transcripts encoding key enzymes involved in fatty acid biosynthesis, namely long-chain fatty acid-CoA ligase, 3-hydroxyacyl-CoA dehydrogenase, enoyl-CoA hydratase/long-chain 3-hydroxyacyl-CoA dehydrogenase, and fatty acid elongase, were found to be upregulated. These enzymes are essential in the early stages of fatty acid biosynthesis, such as desaturation and elongation. This upregulation persisted for the duration of the 4-day cobalt enrichment culture, with fold changes surpassing two, until it reached a plateau at 8 days. Additionally, the expression of pyruvate dehydrogenase, a critical gene responsible for supplying energy and carbon molecules during fatty acid biosynthesis, was found to be increased during initial cobalt enrichment (2 days). Expression profiles of genes involved in fat metabolisms lipids synthesis, and TAG assembly indicated a redirection of free long-chain fatty acids and very long-chain fatty acids away from TAG assembly and oxidation. As a replacement, they functioned as precursors and the foundation for fatty acid-derived hydrocarbons [66]. Moreover, transcriptome analysis revealed the presence of prokaryotic pathways, along with 17 unigenes known as fatty acid synthase (animal type, EC 2.3.1.85). This shows that fatty acid elongation can occur in the cytosol as well as the chloroplasts. Previous studies suggested an alternate TAG synthesis mechanism in yeast, plants, and green microalgae, involving phospholipid:diacylglycerol acyltransferase (PDAT) and the use of phospholipids as donors [66]. Interestingly, the SAG 807-1 transcriptome contained nine PDAT unigenes [66].

Cobalt stress altered the amounts of each hydrocarbon compared to the non-stressed condition (Fig. 6B). Of note was the increase in alkatriene, a compound found in strain A algae, which produces primarily *n*-alkadiene and triene hydrocarbons with C23–C33 odd-carbon-numbered chains. Our finding suggests that cobalt stress influences hydrocarbon composition and leads to an abundance of alkatriene in the algae. The distribution of hydrocarbons is influenced by genetic factors, which lead to strain-specific variations even when cultivated under identical conditions [53]. Dienes, which are characterized by mid-chain unsaturation primarily in a *cis* configuration, are the predominant hydrocarbons [53]. Trienes, which are less frequent, often exhibit two conjugated mid-chain unsaturations; whereas some rare instances imply two conjugated double bonds in a terminal position. Experiments using radio-labeled tracers have revealed that oleic acid serves as the direct precursor for dienes and trienes [60, 61]. Additionally, the decarboxylation of very long-chain fatty acid derivatives, activated by a β-substituent, represents the final step in the pathway leading to terminal unsaturation.

Erlenmeyer flasks resulted in the highest amount of 1,19-eicosadiene (40.17%) relative to the total hydrocarbon content (Fig. 6B); whereas the open raceway pond yielded the maximum concentration of E,E-1,9,17-docasatriene (26.73%). The amount of 13-docosen-1-ol (Z) varied across different cultivation conditions, with the highest concentration (47.77%) observed in the open raceway pond. In comparison, its concentration was only 29.15% and 30.15% in the flask and flat-panel photobioreactor cultivations, respectively (Fig. 14). The preference for hydrocarbons over TAG stems from their high energy density and compatibility with existing petroleum infrastructure [75]. The colonial microalga *B. braunii* is recognized as a highly promising candidate for biofuel production, as its hydrocarbon oils make up 25%–75% of its dry weight. Notably, these hydrocarbons are stored predominantly in the extracellular space, in contrast to most other oleaginous microalgae, which accumulate lipids in the cytoplasm [76]. Through catalytic hydrocracking, hydrocarbon oils can be converted into transport fuels, such as gasoline, kerosene, and diesel [77]. The discovery of *B. braunii* fossils in organic remains of oil shales and petroleum source rocks, along with its hydrocarbon oils in crude oils, indicates the significant contribution of this alga to petroleum generation [78].

**Fig. 7:**
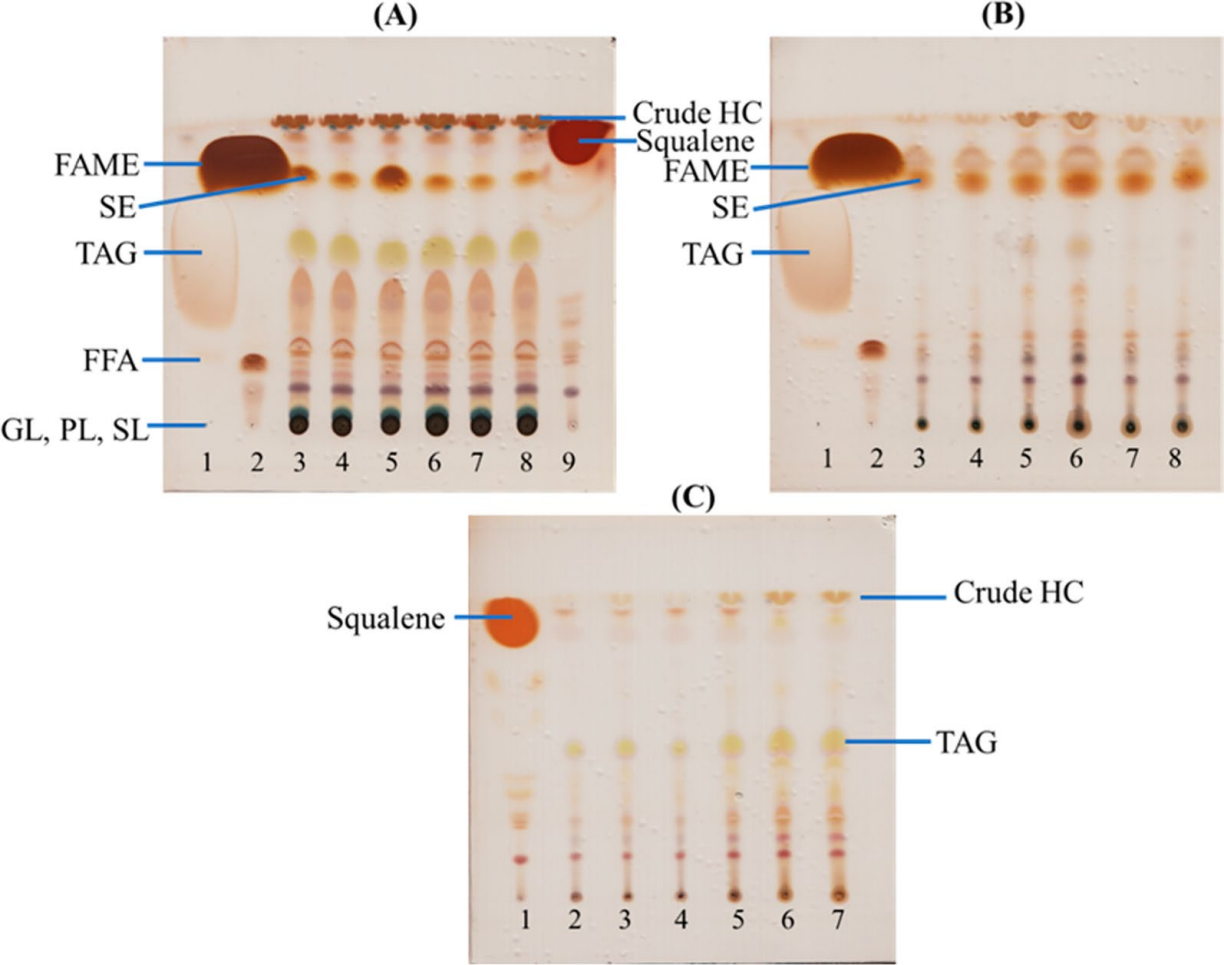
TLC of all extracted crude lipids from *B. braunii* cultivated in Erlenmeyer flasks, flat-panel photobioreactor, and open raceway pond under normal cobalt (lanes 3, 4, 5) and high cobalt (lanes 6, 7, 8). The presence of triacylglycerol (TAG), free fatty acids (FFA), sterols (SE), phospholipids (PL), glycolipids (GL), sphingolipids (SL), and hydrocarbons (HC) including squalene was evaluated in crude lipids (**A**), purified transesterified products (**B**), and crude hydrocarbons (**C**). TAG (lane 1), FAME (lane 2) and squalene (lane 9) were used as standards in panels A and B. In panel C, normal cobalt (lanes 2, 3, 4) and high cobalt (lanes 5, 6, 7) are indicated along with squalene as standard (lane 1)

TLC of the extracted crude lipids from *B. braunii* cultivated in Erlenmeyer flasks, flat-panel photobioreactor, and open raceway pond under normal cobalt (lanes 3, 4, 5 in Fig. 7A) and high cobalt conditions (lanes 6, 7, 8 in Fig. 7A) was carried out to evaluate the presence of TAG, free fatty acids, diacylglycerol, monoacylglycerol, sterols, phospholipids, glycolipids, sphingolipids, and hydrocarbons (including squalene). To capture both non-polar and polar lipids, we used a solvent mixture of *n*-hexane:diethyl ether:acetic acid, whose components had varying polarity indices. For example, *n*-hexane is extremely non-polar (polarity index of 0.1), while acetic acid is more polar (polarity index of 6.2) due to its ionization capacity. It should be noted that the components in the solvent mixture are not present in equal quantities, and both the polarity index and relative amounts of each component determine how they carry specific lipid species up the TLC plate. TLC analysis of the extracted total lipids revealed the presence of squalene and hydrocarbons, which appeared at the top of the chromatogram. To confirm the presence of sterols and TAG, as well as free fatty acids, we performed TLC analysis of FAME (Fig. 7B). The absence of TAG indicated the completion of the transesterification reaction. The hydrocarbons extracted from the various cultivations confirmed the presence of squalene and lipid types (Fig. 7C). Hydrocarbons were much more abundant under cobalt stress (lanes 5, 6, 7 in Fig. 7C) than under normal cobalt conditions (lanes 2, 3, 4 in Fig. 7C). TLC analysis provided insights into the lipid composition of *B. braunii* cultivated under different conditions, highlighting the presence of various lipid classes and hydrocarbons in the extracted crude lipids.

### Estimation of pigments and protein in different cultivations of *B. braunii*

**Fig. 8:**
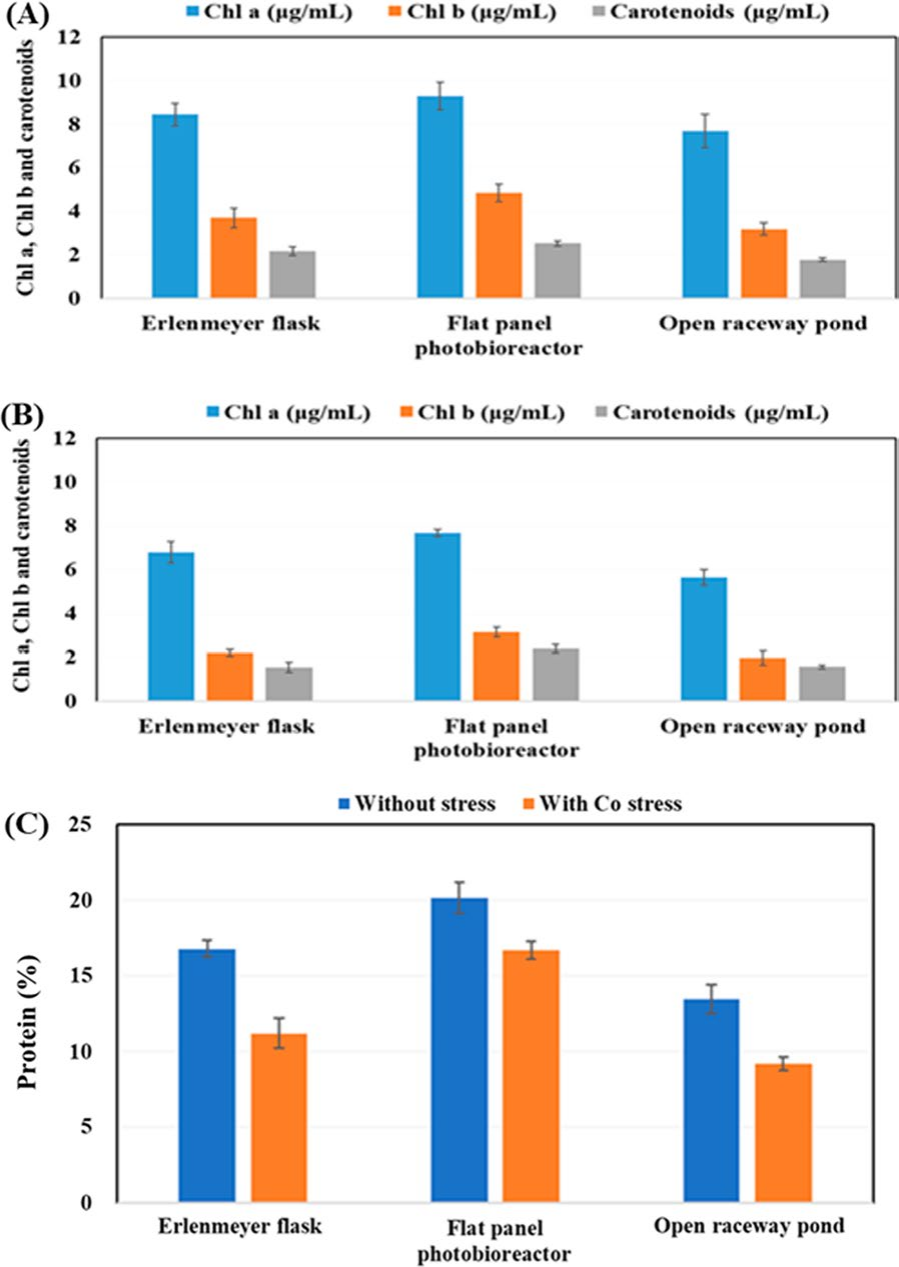
Analysis of pigments (Chl a, Chl b, and carotenoids) extracted from *B. braunii* cultivated under normal cobalt (**A**) and high cobalt (**B**) conditions. Comparison of protein quantity in *B. braunii* cultivated under normal cobalt and high cobalt conditions (**C**)

Microalgae such as *B. braunii* possess two primary categories of photosynthetic pigments: chlorophylls and carotenoids. Notably, in certain *B. braunii* B strains, carotenoids accumulate within the extracellular matrix, suggesting functions other than photosynthesis [79]. Similar to other microalgae, *B braunii* SAG 30.81 possesses Chl a, Chl b, and carotenoids. The levels of Chl a, Chl b, and carotenoids in this strain are influenced by nutrient availability and light intensity. When grown in an Erlenmeyer flask, the alga produced 8.43 ± 0.50 μg/mL Chl a, 3.69 ± 0.45 μg/mL Chl b, and 2.17 ± 0.20 μg/mL carotenoids (Fig. 8A). With a flat-panel photobioreactor, these amounts increased to 9.28 ± 0.64 μg/mL for Chl a, 4.84 ± 0.40 μg/mL for Chl b, and 2.51 μg/mL for carotenoids. This increase may be attributed to more efficient mixing of the culture and aeration during cultivation. However, when the alga was grown in an open raceway pond, the levels decreased to 7.68 ± 0.76 μg/mL for Chl a, 3.18 ± 0.27 μg/mL for Chl b, and 1.77 ± 0.08 μg/mL for carotenoids (Fig. 8A).

When *B. braunii* was cultivated with cobalt nitrate at 5 mg/L in BBM medium, the levels of all pigments showed a decline, reaching a maximum of 7.65 ± 0.65 μg/mL for Chl a, 3.14 ± 0.22 μg/mL for Chl b, and 2.40 ± 0.19 μg/mL for carotenoids in a flat-panel photobioreactor (Fig. 8B). In all cases, the Chl a/Chl b ratio ranged around 1.91–2.41 when cultivated with a normal amount of cobalt, but increased to 2.43–3.07 with a high cobalt concentration. The Chl a/Chl b ratio and carotenoid/chlorophyll ratio are commonly used as indicators to assess the photosynthetic cell response to changes in irradiance, nutrients, temperature, or salinity [80]. *B. braunii* CCAP 807/2 produced 4–6 mg/L Chl a in nitrogen-limited cultures under low light conditions, but less than 2 mg/L from day 9 under high irradiance [81].

The protein content of *B. braunii* varied depending on the cultivation strategy employed (Fig. 8C). The highest protein content (20.15 ± 1.03%) was observed when using a flat-panel photobioreactor, and the lowest with an open raceway pond (13.46 ± 0.93%). Interestingly, the protein content was higher in the normal cobalt condition, decreasing to 11.20 ± 1.0% (Erlenmeyer flask), 16.70 ± 0.56% (flat-panel photobioreactor), and 9.18 ± 0.43% (open raceway pond) when cultivation was carried out under high cobalt. Subagyono et al. suggested that *B. braunii* could synthesize up to 14.8% (wt/wt) of protein [82]. However, using a strain similar to the one employed in this study, the protein content was reported to be only 6.2% and 4.2% under normal and enriched cobalt conditions, respectively [13].

### Thermogravimetric and differential thermogravimetric curves of green biomass, white deposits, and de-oiled biomass

**Fig. 9:**
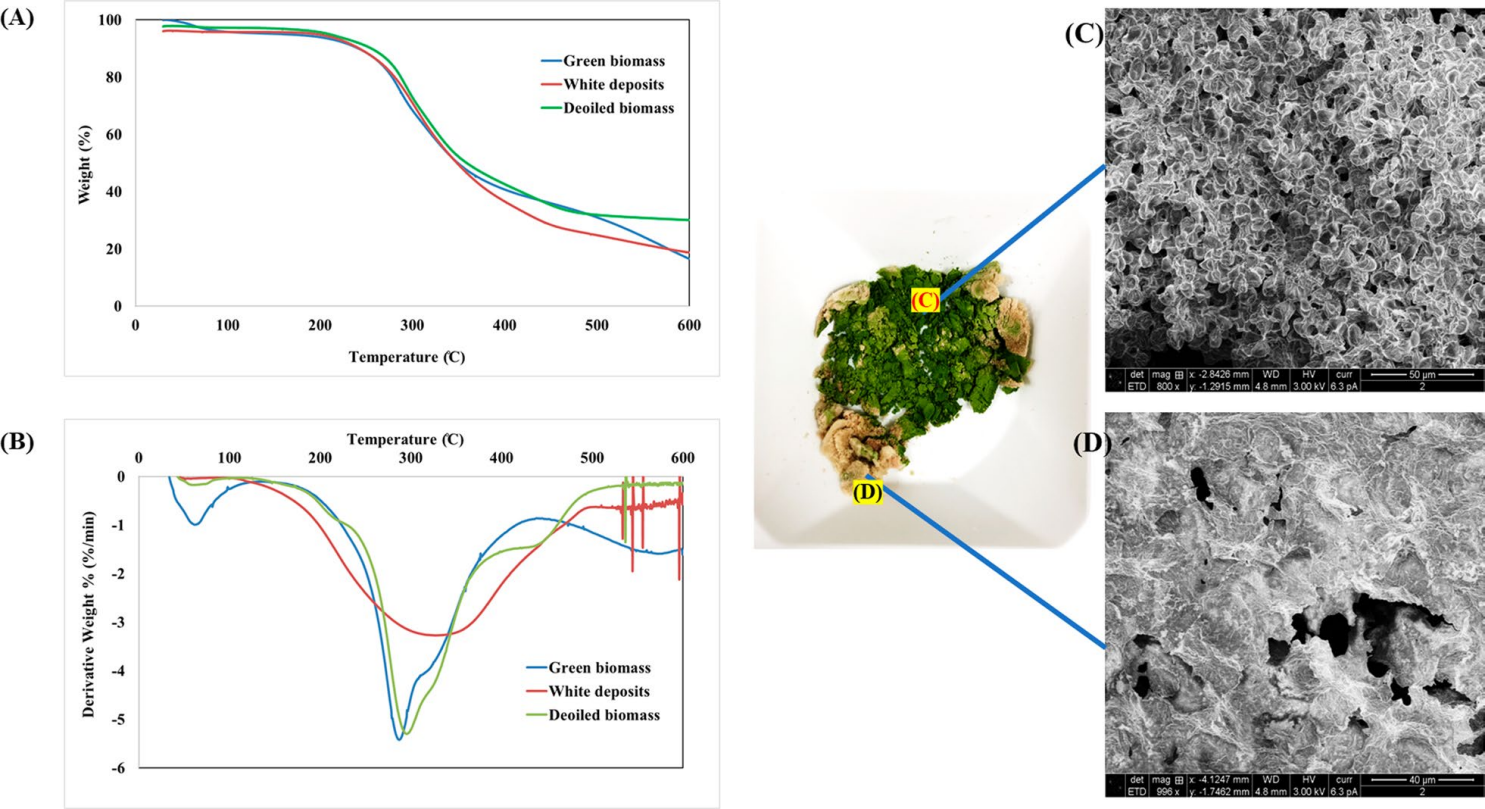
TG (**A**) and DTG (**B**) curves showing the pyrolysis of green microalgal biomass, white deposits, and de-oiled biomass of *B. braunii*. **C**, **D** Scanning electron microscopy images of green biomass and white deposits of freeze-dried cells cultivated under cobalt-rich conditions in a flat-panel photobioreactor

The pyrolysis characteristics both the thermogravimetric curves (TG, in units of wt%) and differential thermogravimetric curves (DTG, in units of wt%/min) of green biomass, white deposits, and de-oiled biomass are shown in Fig. 9. The samples displayed different weight loss profiles, which were divided into three stages. The initial phases take place at 156 °C, where moisture and low-boiling-point organic compounds evaporate depending on the heating rates. While chlorophyll decomposition may have occurred at 80–110 °C, owing to its inherent instability, no typical pyrolysis compounds, e.g., phytane and pristane [83], were detected at this stage. Thermal degradation, depolymerization, decarboxylation, and cracking of carbohydrates, proteins, chlorophyll, and lipid molecules in microalgae happened during active pyrolysis. Carbohydrates disintegrate between 200 and 300 °C, whereas proteins breakdown between 280 and 400 °C. The lipids in the microalgae were thermally destroyed at temperatures ranging from 270 to 550 °C, a range comparable to that reported by Chen et al. [84].

Biomass started to degrade at 40 °C and weight loss was observed. Four distinct degradation temperature zones were noted from 40 to 565 °C. Maximum weight loss occurred at 286 °C, but another unique point occurred at 570 °C. The peak of the first derivative indicates the point of greatest rate of change (i.e., inflection point) on the weight loss curve. De-oiled biomass also started to degrade at 40 °C, although differently from green biomass. Weight loss peaked at 296 °C, followed by another event at 446 °C. The profile matched that reported by Li et al. with hexane-extracted *B. braunii* [85].

Two prominent peaks were found during active pyrolysis in the DTG curve (Fig. 9B). The first, weaker peak, appeared in the temperature range of 150–250 °C and showed protein and carbohydrate breakdown. The second peak, which was more apparent and occurred in the temperature range of 280–350 °C, suggested lipid breakdown. Passive pyrolysis was seen to begin at temperatures ranging from 526 to 557 °C, depending on the heating rate, in the third stage. Flat curves on the graph marked this phase. Compounds decomposed predominantly by gasification and the production of nonvolatile carbon compounds in passive pyrolysis. These nonvolatile carbon molecules then evaporated, producing gaseous CO and CO_2_ at high temperatures [86]. Other main microalgal constituents pyrolyzed or thermally decomposed at lower temperature include chlorophyll (80–145 °C), carbohydrates (200–400 °C), and proteins (220 and 300 °C) [82].

A distinct profile was observed with white deposits of *B. braunii*, with only one strong peak at 315–345 °C (Fig. 9B) due to degradation of long-chain hydrocarbons. This result indicates that white deposits were devoid of protein and chlorophyll. Liu et al. also compared the pyrolytic behavior of dry biomass, de-oiled biomass, and oil extracted from *B. braunii*. They confirmed that the white deposits observed were not lipids, but rather long-chain hydrocarbons, as they exhibited a different profile compared to green biomass [87]. To provide further confirmation, freeze-dried cells cultivated in a cobalt-rich medium were examined by scanning electron microscopy (Fig. 9C, D). Accordingly, the green parts resembled tiny cells, while the white portion appeared as a solid matrix lacking any structural components.

### Pyrolysis of green microalgal biomass, white deposits, and de-oiled biomass by single-shot Py-GC–MS

The pyrolytic behavior of *B. braunii* green biomass, white deposits, and de-oiled biomass was investigated by analytical Py-GC–MS. The molecular distribution of the pyrolysate provided preliminary indications on the possible composition of bio-oil. Green and de-oiled biomass presented a similar molecular pattern, with abundant thermal degradation products of proteins (indole, phenols), carbohydrates (furanmethanol, hydroxycyclopentenone), and lipids (phytadienes from chlorophyll). The pyrograms resembled the one derived from fresh *B. braunii* [88]. Numerous aliphatic compounds were identified in the pyrolysis total ion chromatogram of all biomass samples (Supplementary Fig. 1; Table 4).

**Table 4:** Chemical components obtained from Py-GC–MS analysis of freeze-dried green biomass, white deposits, and de-oiled biomass of *B. braunii*

Peak no.	Green biomass			White deposits			De-oiled biomass		
	Compounds	RT	%	Compounds	RT	%	Compounds	RT	%
1	Methylenecyclopropane	5.76	3.98	1,3-Butadiene	5.79	7.48	2-Butene	5.77	3.60
2	2-Methyl-1-butene	5.86	0.41	Cyclopropane, ethyl-	5.90	0.48	2-Pentene	6.00	1.67
3	2-Pentene	5.99	2.66	2-Pentene	6.03	3.22	Cyclopropane, ethylidene-	6.15	1.59
4	1,4-Pentadiene	6.14	2.00	1,4-Pentadiene	6.18	2.00	1,3-Pentadiene	6.27	1.39
5	1,3-Pentadiene	6.26	1.43	Cyclopropane, ethylidene-	6.29	1.22	Cyclopropylacetylene	6.41	3.52
6	Cyclopropylacetylene	6.41	4.18	Cyclopropylacetylene	6.43	5.49	Cyclohexane	6.57	1.65
7	1-Hexene	6.56	2.30	Cyclohexane	6.58	2.36	Benzene, chloro-	6.68	3.71
8	1,4-Hexadiene	6.67	0.44	3-Hexen-1-ol, (E)-	6.70	0.31	2-Hexyne	7.04	1.16
9	1,4-Hexadiene, (Z)-	6.82	0.95	1,4-Hexadiene, (Z)-	6.84	0.71	1,3-Cyclopentadiene, 5-methyl-	7.30	2.12
10	2-Hexyne	7.03	0.97	2-Propenenitrile	7.05	0.85	1,3-Cyclopentadiene, 5-methyl-	7.38	1.50
11	1,3,5-Hexatriene	7.28	2.83	1,3-Cyclopentadiene, 5-methyl-	7.30	2.25	Cyclopentane, 1,3-dimethyl-	7.57	1.43
12	1,3-Cyclopentadiene, 5-methyl-	7.37	2.03	3-Methylenecyclopentene	7.39	1.54	1,3-Cyclopentadiene, 1-methyl-	7.69	2.08
13	Cyclopentane, 1,2-dimethyl-, cis-	7.56	1.91	Cyclopentane, 1,2-dimethyl-, cis-	7.57	1.88	1,5-Hexadiyne	7.87	3.99
14	1,3-Cyclopentadiene, 1-methyl-	7.67	2.69	1,3-Cyclopentadiene, 1-methyl-	7.70	2.91	Cyclopentane, ethylidene-	8.23	0.67
15	1,5-Hexadiyne	7.85	4.61	Benzene	7.86	7.91	(5-Methylcyclopent-1-enyl)methanol	8.46	0.26
16	Norbornane	8.22	0.72	Vinylcyclopentane	8.23	0.41	1,3-Cyclopentadiene, 1,2-dimethyl-	8.65	0.53
17	Cyclopropane, trimethylmethylene-	8.45	0.40	Bicyclo[4.1.0]hept-2-ene	8.65	0.42	1,3-Cyclopentadiene, 1,2-dimethyl-	8.79	0.34
18	2-Methylcyclohexylamine, *N*-acetyl (stereoisomer 2)	8.52	0.33	1,3-Cyclopentadiene, 1,2-dimethyl-	8.81	0.43	Styrene	8.92	0.81
19	1,3-Cyclopentadiene, 1,2-dimethyl-	8.64	0.72	1-Octene	9.02	1.44	*cis*-1-Butyl-2-methylcyclopropane	9.02	1.11
20	1-Methylcyclohexa-2,4-diene	8.79	0.72	Cyclotrisiloxane, hexamethyl-	9.24	0.99	Toluene	9.49	3.75
21	Cyclopropane, trimethylmethylene-	8.92	0.35	1,3-Cyclopentadiene, 5,5-dimethyl-	9.32	0.24	Pyridine	9.89	0.94
22	1-Octene	9.01	1.67	Toluene	9.47	5.99	Bicyclo[2.1.1]hex-2-ene, 2-ethenyl-	10.19	0.44
23	Bicyclo[4.1.0]hept-2-ene	9.31	0.32	Pyridine	9.83	0.79	2,6-Pyridinedicarboxaldehyde, 3-(phenylmethoxy)-, bis[methyl(2-pyridyl)hydrazone]	10.61	0.80
24	Toluene	9.47	3.70	Bicyclo[2.1.1]hex-2-ene, 2-ethenyl-	10.19	0.40	1-Nonene	10.72	1.08
25	Pyridine	9.85	0.93	Toluene	10.36	0.25	Pyridine, 2-methyl-	11.00	0.58
26	1-Cyclohexene, 1-ethynyl-	10.17	0.52	1-Nonene	10.72	1.49	Ethylbenzene	11.14	2.08
27	Bicyclo[2.2.1]hept-2-en-7-ol	10.34	0.42	Pyridine, 2-methyl-	10.89	0.30	*o*-Xylene	11.27	1.10
28	2-Propenoic acid	10.60	0.79	Ethylbenzene	11.12	2.51	*o*-Xylene	11.80	0.69
29	1-Nonene	10.72	1.43	o-Xylene	11.26	0.97	1,3,5,7-Cyclooctatetraene	11.96	2.64
30	Pyridine, 2-methyl-	10.96	0.36	p-Xylene	11.80	0.59	1-Decene	12.46	0.95
31	Ethylbenzene	11.11	2.10	Styrene	11.95	3.23	Cyclohexane, 1,2-dichloro-	12.52	0.34
32	o-Xylene	11.26	1.40	Isoamyl cyanide	12.31	0.27	1H-Pyrrole, 2-methyl-	12.63	0.33
33	o-Xylene	11.79	0.88	1-Decene	12.45	1.37	1H-Indene, 2,3,4,7-tetrahydro-	12.73	1.18
34	Styrene	11.95	2.52	Bicyclo[7.1.0]decane	12.51	0.36	Benzene, 1-ethyl-3-methyl-	12.85	0.41
35	Cyclodecane	12.46	1.28	1H-Pyrrole, 3-methyl-	12.62	0.29	Benzene, 1,4-dichloro-	13.06	1.06
36	Bicyclo[3.2.1]octan-3-one	12.51	0.44	1H-Indene, 2,3,4,7-tetrahydro-	12.72	0.57	Benzene, (1-methylethyl)-	13.27	0.35
37	Benzene, propyl-	12.72	0.73	Benzene, 1-ethyl-3-methyl-	12.85	0.63	Benzene, 1-ethenyl-3-methyl-	13.74	0.93
38	Benzene, 1-ethyl-3-methyl-	12.85	0.62	Benzene, 1-ethyl-4-methyl-	13.28	0.42	Benzene, 1-ethenyl-2-methyl-	13.81	0.31
39	Pyridine, 2,4-dimethyl-	13.01	0.32	Benzene, 1-ethenyl-2-methyl-	13.73	1.05	Benzene, 1,4-dichloro-	13.87	0.55
40	Benzene, (1-methylethyl)-	13.26	0.32	Indane	13.81	0.37	1H-Indene, 1-chloro-2,3-dihydro-	14.04	0.92
41	Mesitylene	13.50	0.46	1-Undecanol	14.10	1.55	1-Undecanol	14.11	1.02
42	Benzene, 2-propenyl-	13.73	1.09	1,10-Undecadiene	14.16	0.31	Benzene, 2-propenyl-	14.32	0.29
43	2-Cyclohexenol, 4-acetamido-, E\\trans-	14.01	0.41	Deltacyclene	14.32	0.39	Benzene, cyclopropyl-	14.40	0.58
44	Formic acid, undecyl ester	14.11	1.48	Cyclopentasiloxane, decamethyl-	14.41	0.80	2-Chlorostyrene	14.54	0.29
45	1,10-Undecadiene	14.16	0.40	Benzene, 1-ethynyl-4-methyl-	14.89	1.73	Benzene, 1-propynyl-	14.90	2.06
46	(Z)-1-Phenylpropene	14.31	0.52	Tricyclo[3.2.2.0(2,4)]non-8-ene-6-carboxylic acid,7-[(phenylamino)carbonyl]-	14.96	0.31	Benzonitrile	15.09	0.70
47	3-Phenylpropanol	14.40	0.85	Benzonitrile	15.07	0.37	3-Oxaspiro[5.5]undecane-1,5-dione, 4-methyl-3-phenyl-	15.32	0.88
48	Benzene, 1-ethynyl-4-methyl-	14.88	2.52	Benzene, 2-ethenyl-1,4-dimethyl-	15.32	0.32	Cyclododecane	15.66	0.61
49	Hexahydro-1H-pyrrolizin-7a-ylmethanamine	15.30	0.81	Cyclododecane	15.65	0.93	11,14-Eicosadienoic acid, methyl ester	15.72	0.57
50	Cyclododecane	15.65	0.84	1,11-Dodecadiene	15.71	0.45	Phenol	16.00	1.60
51	1,11-Dodecadiene	15.71	0.49	Phenol	15.98	1.23	2-Methylindene	16.50	1.23
52	Phenol	15.98	1.12	2-Methylindene	16.49	1.07	Naphthalene, 1,2-dihydro-	16.72	1.39
53	2-Methylindene	16.50	1.75	Naphthalene, 1,2-dihydro-	16.71	0.99	2-Methylindene	16.79	0.62
54	2-Methylindene	16.70	1.75	Benzene, (1-methyl-2-cyclopropen-1-yl)-	16.78	0.46	Naphthalene	17.04	0.35
55	2-Methylindene	16.78	0.70	Naphthalene	17.03	0.34	1-Tridecene	17.10	0.78
56	1-Tridecene	17.10	0.73	1-Tridecene	17.09	0.76	Naphthalene	17.24	4.43
57	p-Cresol	17.23	1.17	Phenol, 3-methyl-	17.23	0.57	Naphthalene	17.48	1.62
58	Naphthalene	17.47	1.87	Naphthalene	17.46	1.71	Oxacyclotetradecane-2,11-dione, 13-methyl-	17.87	0.39
59	Cyclohexanecarboxylic acid, undec-10-enyl ester	17.86	0.42	Benzyl nitrile	17.99	0.87	Benzonitrile, 2-methyl-	18.01	0.94
60	Benzyl nitrile	17.98	0.64	1H-Cyclopropa[b]naphthalene, 1a,2,7,7a-tetrahydro-	18.17	0.42	1-Methyl-1-ethoxy-1-silacyclopentane	18.17	0.93
61	Naphthalene, 1,2-dihydro-3-methyl-	18.16	0.58	Naphthalene, 1,2-dihydro-4-methyl-	18.29	0.29	Cyclotetradecane	18.45	0.67
62	Cyclotetradecane	18.45	1.07	Cyclotetradecane	18.45	1.06	2,6-Dimethylbicyclo[3.2.1]octane	18.51	0.51
63	1,11-Dodecadiene	18.51	0.65	1,11-Dodecadiene	18.51	0.54	Quinoline	18.77	0.39
64	Resorcinol, 2TMS derivative	18.60	0.33	Isoquinolinium, 2-methyl-, iodide	18.74	0.45	Naphthalene, 1-methyl-	18.91	0.27
65	Isoquinolinium, 2-methyl-, iodide	18.75	0.64	Naphthalene, 1-methyl-	19.02	0.58	Naphthalene, 2-methyl-	19.03	0.52
66	Naphthalene, 2-methyl-	19.02	0.89	1H-Indene, 1-ethylidene-	19.29	0.63	l-Leucine, *N*-dimethylaminomethylene-	19.20	0.56
67	4,8,12-Trimethyltridecan-4-olide	19.15	0.54	N-Acetyltyramine	19.66	0.38	Benzocycloheptatriene	19.30	0.65
68	Benzocycloheptatriene	19.29	0.80	Cetene	19.71	0.71	Cyclopentadecane	19.72	1.35
69	Tyrosol, acetate	19.66	0.71	1,11-Dodecadiene	19.78	0.26	Biphenyl	20.07	0.36
70	1-Pentadecene	19.71	0.83	Naphthalene, 1,3-dimethyl-	20.28	0.38	Naphthalene, 2-chloro-	20.26	1.75
71	1,11-Dodecadiene	19.78	0.36	Indole	20.72	1.68	Indole	20.73	1.67
72	Biphenyl	20.07	0.37	Cetene	20.91	0.61	2-Bromopropionic acid, tridecyl ester	20.92	0.78
73	Naphthalene, 2-ethyl-	20.27	0.39	[1-(Morpholin-4-yl)cyclopentyl]methanamine	20.97	0.45	1,13-Tetradecadiene	20.98	0.53
74	9-Decenoic acid	20.36	0.35	5,8,11-Eicosatriynoic acid, TMS derivative	21.20	0.28	Acenaphthylene	21.51	1.05
75	Indole	20.73	1.84	Acenaphthylene	21.52	0.25	Indole, 3-methyl-	21.68	1.33
76	1,7-Hexadecadiene	20.85	0.41	1H-Indole, 2-methyl-	21.66	0.52	1-Heptadecene	22.05	0.36
77	1-Octadecanol	20.91	0.81	8-Heptadecene	22.04	0.55	Naphthalene, 1,4-dichloro-	22.84	0.36
78	11-Hexadecen-1-ol, acetate, (Z)-	20.97	0.74	Orcinol	22.22	0.23	1H-Phenalene	23.08	0.37
79	Acenaphthylene	21.52	0.54	Benzonitrile, 2,4,6-trimethyl-	22.72	0.27	1-Octadecanol	23.16	0.67
80	1H-Indole, 2-methyl-	21.67	0.93	Oxirane, hexadecyl-	23.08	0.25	Ethanol, 2-(9-octadecenyloxy)-, (Z)-	23.24	0.30
81	8-Heptadecene	21.89	0.31	1-Octadecanol	23.16	0.70	2H-Indol-2-one, 1,3-dihydro-	23.49	0.38
82	8-Heptadecene	22.04	0.56	1-Nonadecene	24.32	0.48	12-Dimethylamino-10-oxododecanoic acid	23.74	0.39
83	1,15-Pentadecanediol	22.11	0.32	Methyl glycocholate, 3TMS derivative	24.65	0.38	Octadecanal	24.22	0.26
84	Benzonitrile, 2,4,6-trimethyl-	22.72	0.41	1-Docosene	25.57	0.42	1-Nonadecene	24.32	0.38
85	Papaveroline, 1,2,3,4-tetrahydro-	22.93	0.53	Phenanthrene	26.21	0.23	Benzeneacetonitrile, 3-hydroxy-	24.73	0.67
86	8-Dodecen-1-ol, acetate, (Z)-	23.07	0.45	8,14-Seco-3,19-epoxyandrostane-8,14-dione, 17-acetoxy-3á-methoxy-4,4-dimethyl-	26.59	0.30	Melezitose	25.05	0.64
87	1-Octadecanol	23.16	0.91	17-Pentatriacontene	26.87	0.26	Fumaric acid, 2-ethylhexyl dodec-2-en-1-yl ester	25.47	0.34
88	11-Hexadecen-1-ol, acetate, (Z)-	23.23	0.47	1-Tricosene	27.00	0.40	1-Hexacosene	25.58	0.63
89	Ethanol, 2-(9-octadecenyloxy)-, (Z)-	24.21	0.32	Ethanol, 2-(9-octadecenyloxy)-, (Z)-	27.12	0.24	Anthracene	26.23	1.25
90	9-Nonadecene	24.32	0.60	*N*-(2-Benzyl-3-isopropyl-cyclopentylidene)-*N*'-(3,5- dinitrophenyl)-hydrazine	27.53	0.28	*n*-Hexadecanoic acid	27.73	0.35
91	Isoindolo[2,1-a]quinazoline-5,11-dione, 6- (dimethylamino)-6,6a-dihydro-	24.69	0.41	17-Pentatriacontene	28.53	0.25	Fumaric acid, 2-ethylhexyl tridec-2-yn-1-yl ester	27.94	0.28
92	á-D-Glucopyranose, 1,6-anhydro-	24.83	0.35	1-Tetracosene	28.68	0.53	1-Hexacosene	28.69	0.32
93	11-Eicosenol	25.45	0.38	1-Docosene	30.75	0.36	Anthracene, 1-chloro-	29.91	0.49
94	Behenic alcohol	25.57	0.57	Octadecane, 3-ethyl-5-(2-ethylbutyl)-	33.06	0.31	Benzene, 1-chloro-3-(phenylethynyl)-	30.24	0.69
95	1-Heneicosyl formate	27.00	0.38	1-Hexacosene	33.32	0.62	Octadecane, 3-ethyl-5-(2-ethylbutyl)-	30.54	0.28
96	1-Tetracosene	28.69	0.47	9-Octadecenamide, (Z)-	35.31	0.35	Pyrene	32.49	0.46
97	1-Heneicosyl formate	30.74	0.34	Octadecane, 3-ethyl-5-(2-ethylbutyl)-	36.25	0.40	17-Pentatriacontene	33.33	0.26
98	Tetracosan-10-yl acetate	33.33	0.35	17-Pentatriacontene	36.59	0.54	Pyrene	34.14	0.60
99	9H-Pyrido[3,4-b]indole	35.42	0.32	1-Hexacosanol	40.78	0.28	9H-Pyrido[3,4-b]indole	35.61	0.52
100	1-Hexacosene	40.79	0.36	9-Octadecenamide, (Z)-	43.85	0.28	17-Pentatriacontene	36.61	0.53

Retention times (RT) are indicated

The pyrolyzed products were similar to those identified in other algae [89]. The abundance of hydrocarbons and aromatic compounds in all samples of *B. braunii* makes this alga an ideal candidate for bio-oil production. The main cycloalkanes were methylenecyclopropane, cyclopropane ethyl, cyclohexane, cyclopentane 1,2-dimethyl-cis-, cyclopropane trimethylmethylene, cis-1-butyl-2-methylcyclopropane, cyclotrisiloxane hexamethyl, cyclodecane, and cyclotetradecane (Table 1). The other most abundant products were alkenes, such as 2-pentene, 1,3-butadiene, 2-butene, 1,4-pentadiene, 1-hexene, 1,4-hexadiene, cyclopropane ethylidene, 1,3,5-hexatriene, 1,3-cyclopentadiene 1-methyl, 1-octene, 1-nonene, 1-decene, 2-methylindene, 1-tridecene, 1,11-dodecadiene, 8-heptadecene, 1-docosene, 17-pentatriacontene, and 1-tricosene. Alkadienes were identified previously in *B. braunii* strain A [90], providing valuable insights into the specific composition of hydrocarbons produced by this algal strain. The white deposits obtained from *Botryococcus* biomass were found to consist mainly of hydrocarbons, as evidenced by the abundance of 1,3-butadiene (C4H6) after pyrolysis. In industrial settings, butadiene is typically produced as a byproduct of steam cracking during the manufacturing of ethylene and other olefins. In this process, aliphatic hydrocarbons are mixed with steam and subjected to high temperatures (often exceeding 900 °C), resulting in the release of hydrogen and the formation of a complex mixture of unsaturated hydrocarbons, including butadiene [91]. The quantity of butadiene depends on the specific hydrocarbons used as feedstock. 2-Propenenitrile, pyridine, indole, toluene, styrene, phenol, and benzonitrile are important protein-derived nitrogen-containing compounds (Table 4). In microalgae, nitrogen-containing compounds and fatty acids derive from proteins and lipids [92]. Nitrogen-containing species, including nitrile, pyrrole, indole, pyridine, amines, and amides, are found in bio-oil. Notably, their content increases during co-pyrolysis, owing to protein decarboxylation, deamination, and Maillard reactions between carbohydrates and proteins [93].

## Conclusion

In conclusion, this study aimed to optimize the biomass production of *Botryococcus braunii* for the maximum utilization of its value-added products such as hydrocarbons, lipids, proteins, and pigments. The research involved various cultivation strategies and assessed the effects of different factors on biomass, hydrocarbon, and lipid production. Initially, the study focused on optimizing the biomass, hydrocarbons, and lipids through different medium compositions. Subsequently, the effects of light intensities and duration were investigated using a multicultivator under photoautotrophic cultivation. Notably, this study is the first to report on the fatty acid and hydrocarbon profiles of *B. braunii* under cobalt treatment. The research found that the cultivation of *Botryococcus* with a high concentration of cobalt (up to 5 mg of cobalt nitrate) resulted in altered hydrocarbon synthesis, including increased levels of n-alkadienes and trienes, as well as lipids containing higher amounts of monounsaturated fatty acids. Importantly, the addition of cobalt did not significantly compromise biomass production. Furthermore, the study explored the scale-up of cultivation up to a 20-L open raceway pond, analyzing its impact on biomass, lipid, and hydrocarbon productivity. The results provided valuable insights into the scalability of the cultivation process. Finally, the study proposed and assessed a new workflow for biofuel generation from dried microalgae biomass. This workflow involved lipid extraction and the pyrolysis of both the whole biomass and lipid-free microalgal residues. The potential for bio-oil production was evaluated as part of this process, along with the production of proteins from the de-oiled biomass. Overall, this study contributes to the understanding of cultivation strategies for optimizing the biomass production of *B. braunii* and its value-added products. The findings provide valuable insights into the effects of cobalt treatment, cultivation scale-up, and the potential utilization of dried microalgae biomass for biofuel generation.

## Supplementary Information

Supplementary Material 1.

## Data Availability

No datasets were generated or analysed during the current study.
