# Supplementary material for: A bioprocess engineering approach for the production of hydrocarbons and fatty acids from green microalga under high cobalt concentration as the feedstock of high-grade biofuels
Source: Biotechnol Biofuels Bioprod. 2024 May 10;17:64. doi: 10.1186/s13068-024-02512-6 (PMC11636930; doi:10.1186/s13068-024-02512-6)

**Supplementary materials**

**Supplementary Figure 1.** Py-GC-MS chromatograms of green biomass, white deposits, and de-oiled biomass from *B. braunii*. Refer to Table 4 for peak assignments.


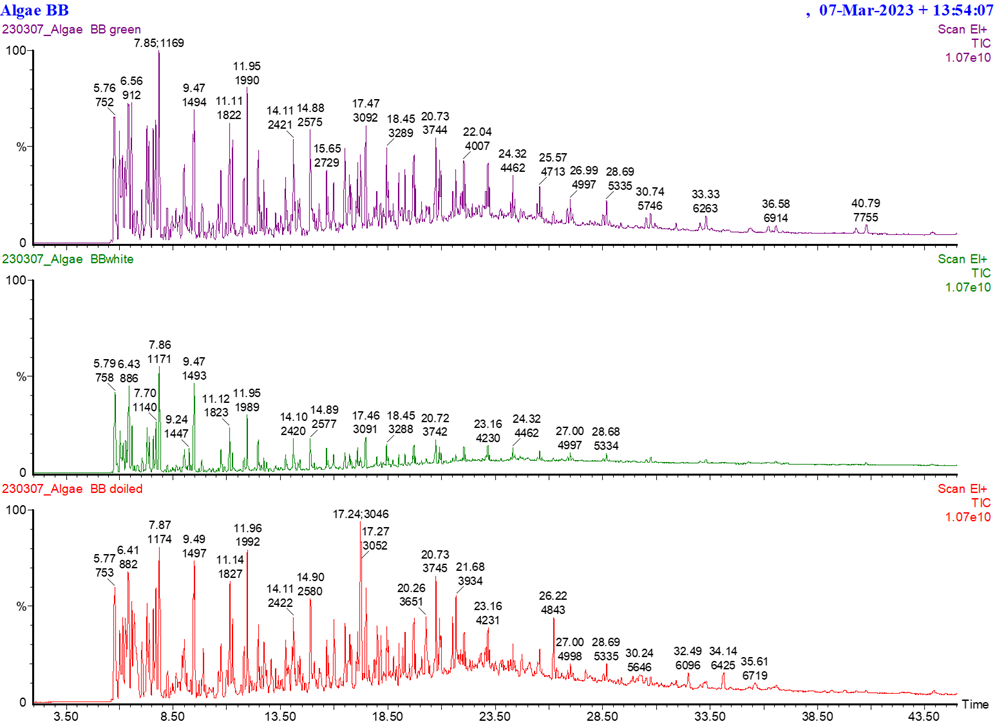

Supplement: Supplementary file 1 — Supplementary Material 1. [file 13068_2024_2512_MOESM1_ESM.docx]
